# Diagnosing scientific replicability through probabilistic distinguishability

**DOI:** 10.1093/bioinformatics/btag140

**Published:** 2026-03-23

**Authors:** Peng Wang, Hongyuan Cao, Xiaoquan Wen

**Affiliations:** School of Mathematics, Jilin University, Changchun, Jilin 130012, China; Department of Statistics and Data Science, Mohamed bin Zayed University of Artificial Intelligence, Abu Dhabi, P.O. Box 7909, United Arab Emirates; Department of Statistics and Data Science, Mohamed bin Zayed University of Artificial Intelligence, Abu Dhabi, P.O. Box 7909, United Arab Emirates; Department of Statistics, Florida State University, Tallahassee, FL 32306, United States; Department of Biostatistics, University of Michigan, Ann Arbor, MI 48109, United States

## Abstract

**Motivation:**

Despite the widely recognized importance of replicability in biological research, computational methods to quantify irreplicability and identify irreplicable instances remain underdeveloped. This article presents an efficient and robust computational framework to address this gap.

**Results:**

To tackle the challenge of defining an acceptable level of intrinsic heterogeneity among replicable studies, we introduce a distinguishability criterion, ensuring that replicable effects, while potentially heterogeneous, can be distinguished from zero effects and maintain consistent directions with high probability. We implement a Bayesian model criticism approach, reporting a Bayesian *P*-value to identify potential irreplicable instances. Through numerical experiments, we demonstrate the efficacy of the proposed methods in detecting batch effects in high-throughput experiments and identifying instances of the publication bias. Finally, we apply the framework to multi-tissue eQTL data from the GTEx consortium, uncovering tissue-specific eQTLs that represent biological heterogeneity across tissues.

**Availability and implementation:**

An R package DiscRep implementing our method is available on GitHub (https://github.com/PengWang96/DiscRep).

## 1 Introduction

Replicability—the ability to consistently reproduce the same findings across repeated experiments—is a cornerstone of scientific research and essential for validating discoveries and building a reliable body of knowledge (Begley and Ellis 2012, Goodman et al. 2016). However, with the growing reliance on complex, high-dimensional data, assessing replicability has become increasingly challenging. Variability across studies, differences in experimental conditions, and data heterogeneity often confound efforts to determine whether findings genuinely replicate.

The importance of replicability assessment extends well beyond evaluating repeated experiments. Multi-tissue expression quantitative trait loci (eQTL) analyses can be naturally framed as evaluating the consistency/replicability of a genetic variant’s effect on a target gene across multiple tissues (Flutre et al. 2013). An effect can be heterogeneous across tissues. Cross-tissue inconsistency may reflect biologically meaningful tissue-specific regulation. Likewise, evaluating gene–environment interactions or spatially variable gene expression patterns across contexts often reduces to testing whether biological effects replicate across different contexts (Li et al. 2022, 2024).

Despite recent progress, two fundamental challenges remain unresolved. The first is conceptual: how to define an acceptable level of heterogeneity among studies considered replicable. While heterogeneity is intrinsic to most scientific measurements (Dekkers 2018, Bogomolov and Heller 2023, McKenzie and Veroniki 2024), there is no consensus on how much variation is permissible. The fixed-effect assumption remains widely used despite its well-recognized limitations (Patil et al. 2016). Some have proposed using directional consistency as a relaxed criterion for replicability (Zhao et al. 2020), but this can be overly lenient and fails to capture consequential quantitative discrepancies.

The second challenge is to implement appropriate statistical methods for quantitatively evaluating irreplicable results across multiple experiments. In practice, inappropriate statistical practice, such as treating a lack of consistently significant *P*-values as direct evidence of irreplicability, can lead to misleading conclusions. Principled statistical approaches for properly evaluating replicability include model comparison, predictive interval checking, and Bayesian model criticism. Model comparison (Li et al. 2011, Wen 2014, Zhao et al. 2020) compares competing statistical models that represent replicable versus irreplicable scenarios and identifies the model most strongly supported by the observed data. Predictive interval checking (Seymour 1993, IntHout et al. 2016, Patil et al. 2016) examines whether predictions generated from a fitted model based on existing experiments fall within an acceptable range of outcomes observed in new replication studies. In this article, we focus on Bayesian model criticism methods (Meng 1994, Gelman et al. 1996, Zhao and Wen 2021). For replicability assessment, a reference model that explicitly characterizes the replicable scenario is specified, and its inadequate fit to the observed data, typically summarized by a small Bayesian *P*-value, is interpreted as evidence against replicability. Like frequentist *P*-values, Bayesian *P*-values provide a calibrated measure of model discrepancy by assessing how surprising the observed data are under the fitted reference model. But unlike frequentist *P*-values, they need not be uniformly distributed when the reference model is true (Meng 1994).

In this article, we introduce DiscRep, a Bayesian framework for assessing replicability using a distinguishability criterion, to address above challenges. Rather than assuming homogeneity or relying on indirect heuristics, DiscRep quantifies whether observed effects are probabilistically distinguishable from null effects under an acceptable level of heterogeneity. This approach provides a principled, context-agnostic, and interpretable measure of replicability that is robust to batch effects (Cochran 1950, Leek et al. 2010), publication bias (Begg and Mazumdar 1994, Egger et al. 1997, Lin and Chu 2018), and other sources of variability. DiscRep leverages posterior predictive checks to detect irreplicable findings and can be easily adapted to diverse data types and experimental designs. We validate the proposed criterion through comprehensive simulation studies and applications to real-world meta-analyses. Our results demonstrate its effectiveness in detecting batch effects, publication bias, and in identifying cross-tissue heterogeneity in eQTL effects. The distinguishability criterion provides a sensitive and reliable tool for assessing replicability, helping to identify robust scientific findings and supporting more accurate interpretations in high-throughput biological research.

## 2 Materials and methods

### 2.1 Distinguishability criterion

Genuine effects are expected to be consistent across multiple replication studies, although some degree of heterogeneity among replications is inevitable (Higgins and Thompson 2002, Higgins 2008). Characterizing this expected heterogeneity is essential for defining replicability. We argue that variations among realizations of a genuine replicable effect are permissible as long as, with high probability, they remain distinguishable from realizations of the reference null effect under the same assumed heterogeneity. We refer to this characterization as the *distinguishability criterion* for defining heterogeneity.

To implement the distinguishability criterion in a statistical model, we consider a replicated realization of a genuine effect, β, derived from an underlying true effect $\bar{\beta }$. Under the commonly assumed normal model, the heterogeneity of the replications can be quantified by a variance parameter, $\phi^{2}$, such that,


(1) $$\beta |\bar{\beta }∼N(\bar{\beta },\phi^{2}).$$

A key challenge in defining a reasonable heterogeneity constraint for replicable signals is that the scale of $\phi^{2}$ depends on the measurement scale of the effect size and ranges from 0 to infinity, making its interpretation context-dependent. The distinguishability criterion addresses this issue by framing $\phi^{2}$ in a classification problem, in which we attempt to distinguish β from the replications of a null reference effect with the same level of intrinsic heterogeneity. The distinguishability criterion states that the probability of misclassifying a realization of a replicable effect as a null effect should be low. Specifically, the misclassification probability is computed as


(2) $$\begin{array}{c} P_{\text{mis}}\left(\bar{\beta },\phi^{2}\right)=P(\text{an}\;\text{effect}\;\text{is}\;\text{misclassified}\;\text{from}\;\text{its}\;\text{generative}\;\text{model})\\ =\int_{-\infty }^{\infty }\frac{N(\beta ;\bar{\beta },\phi^{2})N(\beta ;0,\phi^{2})}{N(\beta ;\bar{\beta },\phi^{2})+N(\beta ;0,\phi^{2})}d\beta \\ =E_{Z}\left[{\left(1+\text{exp}\;\left[\frac{1}{2}{\left(\frac{\left|\bar{\beta }\right|}{\phi }\right)}^{2}+\frac{\left|\bar{\beta }\right|}{\phi }Z\right]\right)}^{-1}\right], \end{array}$$

where *Z* is a standard normal random variable and the notation $N(x;\mu ,\sigma^{2})$ denotes the density function of a normal distribution, $N(\mu ,\sigma^{2})$, evaluated at *x*. The derivation and monotonicity of (2) is provided in Section C.1 and C.2 of the Supplementary Notes, available as supplementary data at *Bioinformatics* online, respectively. Let $k:=\phi /|\bar{\beta }|$. Note that Equation (1) is now expressed as $\beta |\bar{\beta }∼N(\bar{\beta },k^{2}{\bar{\beta }}^{2}),$ where the variance is a function of the mean. The misclassification probability becomes a univariate function of *k*, i.e.


(3) $$P_{\text{mis}}(k)=E_{Z}\left[{\left(1+\text{exp}\;\left[\frac{1}{2}{\left(\frac{1}{k}\right)}^{2}+\frac{1}{k}Z\right]\right)}^{-1}\right].$$

Therefore, to define an upper bound on acceptable heterogeneity of replicable signals, restricting $P_{\text{mis}}$ is equivalent to constraining $\phi^{2}$ (or equivalently *k*) given $\bar{\beta }$. Most importantly, because $P_{\text{mis}}$ is defined on a probability scale, it provides a universally interpretable criterion across different application contexts. For a given $\bar{\beta }$, in the extreme case of minimal heterogeneity, where $\phi^{2}\to 0$, the corresponding $P_{\text{mis}}$ also approaches 0. Conversely, in the case of maximal heterogeneity, where $\phi^{2}\to \infty$, $P_{\text{mis}}$ approaches 0.5, effectively rendering classification equivalent to a random guess. By bounding $P_{\text{mis}}$, the distinguishability criterion ensures that replicated effects remain distinguishable from the null with high probability (see Sections C.1 of the Supplementary Notes, available as supplementary data at *Bioinformatics* online, for derivation). As a consequence, the directions of the replicate effects are also consistent with high probability (see Sections C.3 of the Supplementary Notes, available as supplementary data at *Bioinformatics* online, for derivation). Within this framework, we define an effect as *replicable* if the study-level effects satisfy the criterion at the chosen tolerance level $P_{\text{mis}}$. This formulation permits effect sizes to vary across studies, thereby accommodating an acceptable degree of intrinsic heterogeneity.

### 2.2 Model for replicable effect and posterior predictive checking

We further extend (1) to model observed effects, along with the corresponding standard errors. The resulting hierarchical model, incorporating a user-defined $P_{\text{mis}}$ threshold, fully characterizes the properties of replicable signals given a group of estimated effects and their corresponding standard errors. Henceforth, we refer to this model as the *reference replicability model*.

For a given pre-specified heterogeneity level of replicable signals, defined by $P_{\text{mis}}$, we can uniquely determine the corresponding *k* value. We consider the null (zero) effect as a natural reference point to construct a general reference replicability model implementing distinguishability criterion under model criticism. The reference replicability model, $R_{0}$, is a probabilistic generative model defined as:


(4) $$\bar{\beta }∼1,\beta_{j}|\bar{\beta },k∼N(\bar{\beta },k^{2}{\bar{\beta }}^{2}),{\hat{\beta }}_{j}|\beta_{j}∼N(\beta_{j},{\hat{\sigma }}_{j}^{2}),\;j=1,\ldots ,m,$$

where $\bar{\beta }$ is the grand effect (with a flat prior), $\beta_{j}$ is the underlying true effect in each experiment, ${\hat{\beta }}_{j}$ is the observed effect, and ${\hat{\sigma }}_{j}^{2}$ is the observed variance of effect. The number of experiments/studies is *m*. This implementation assumes a flat prior for $\bar{\beta }$.

To utilize the distinguishability criterion, by default, we sample $-{\;\text{log}\;}_{10}(P_{\text{mis}})$ from a $\text{Uniform}[-{\;\text{log}\;}_{10}(0.05),10]$ distribution and obtain $k=f^{-1}\left(P_{\text{mis}}\right),$ where $f^{-1}$ denotes the inverse mapping of function (3). As *f* is continuous and increases monotonically, small $P_{\text{mis}}$ results in small k, the heterogeneity level. We set $P_{\text{mis}}$ to be in the interval (0,0.05), ensuring a low misclassification probability.

To assess the replicability of a given set of observations, we employ an established statistical approach known as Bayesian posterior predictive checking. Specifically, we fit the reference replicability model to the observed data and identify a notably poor fit of the dataset as a whole. A notably poor fit suggests the presence of observations that deviate from the reference replicability model $R_{0}$. The goodness-of-fit (or lack thereof) is quantified by a Bayesian *P*-value (Gelman et al. 1996), which we refer to as the posterior-predictive replication *P*-value (posterior-PRP).

Let $x^{\mathrm{obs}}=\{({\hat{\beta }}_{j},{\hat{\sigma }}_{j}):j=1,\ldots ,m\}$ and $x^{\mathrm{rep}}$ denote the observed data and potential future observations, respectively. The posterior-PRP is a valid posterior probability defined by


(5) $$\begin{array}{c} P\left(T(x^{\mathrm{rep}})\geq T(x^{\mathrm{obs}})|x^{\mathrm{obs}},R_{0}\right), \end{array}$$

where T(·) is a user-defined test statistic.

Compared to frequentist *P*-values that follow a Uniform(0,1) under the reference replicability model, Bayesian posterior predictive *P*-values are not necessarily uniformly distributed. This is because the Bayesian *P*-value accounts for the uncertainty of nuisance parameters through marginalization rather than relying on pivotal test statistics or asymptotic null distributions (Rubin 1984, Gelman et al. 1996, Gelman 2013) The theoretical properties of the Bayesian *P*-values are studied in detail by Meng (1994), who show, e.g. the distribution of Bayesian *P*-values under the reference replicability model is symmetric on [0,1] and centered at 0.5. Similarly to interpreting traditional *P*-values, a small posterior-PRP suggests that the observed data are unlikely to have arisen from $R_{0}$. Such departures may reflect technical irreplicability (e.g. batch effects, noise) or genuine biological heterogeneity across contexts (e.g. tissue-specific regulation). In our numerical examples, we illustrate how different choices of test statistics can be used to target distinct sources of irreplicable signals.

### 2.3 Detecting different sources of irreplicability

**Figure 1:**
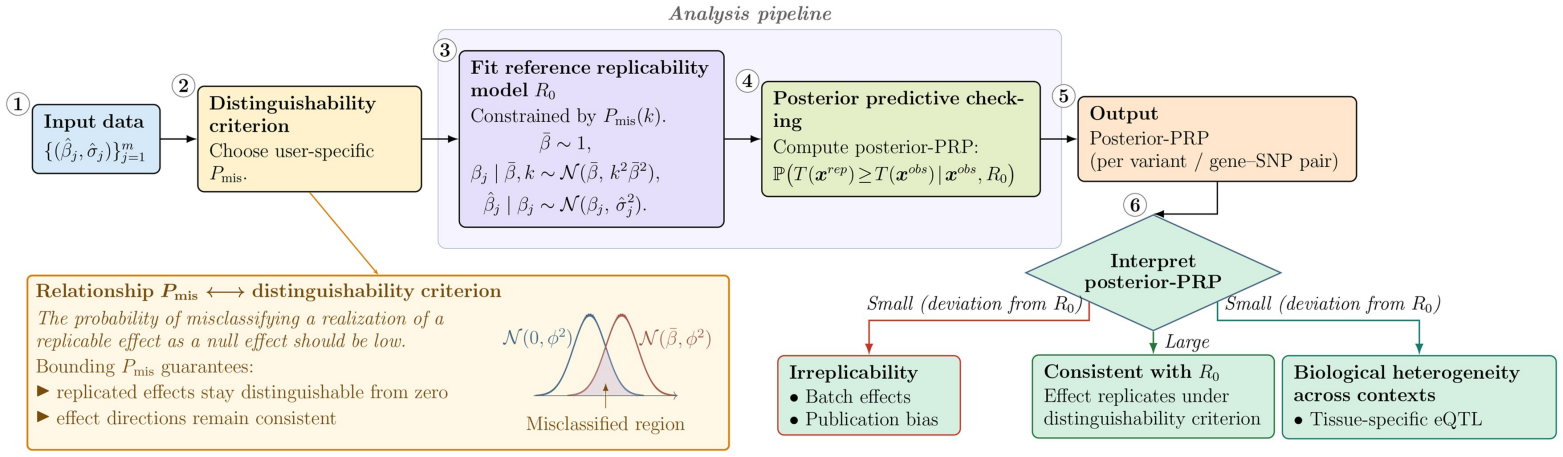
DiscRep workflow.

In practice, irreplicability arises from various factors (e.g. batch effects, publication bias), each leaving distinct footprints in observed data. Designing test statistics that target different sources of irreplicability enhances detection ability (Cochran 1950, Begg and Mazumdar 1994, Egger et al. 1997, Johnson et al. 2007, Lin and Chu 2018). Compared to existing methods, our approach offers unique advantages and greater flexibility in customizing test statistics for this purpose. Posterior predictive checking fundamentally tests variability in observed data, akin to a variance test, while treating $\bar{\beta }$ as a nuisance parameter. Frequentist tests typically replace nuisance parameters with point estimates, disregarding their inherent uncertainty and making them sensitive to outliers. In contrast, the computation of posterior-PRPs integrates out possible $\bar{\beta }$ values from the posterior distribution, fully accounting for the uncertainty of the nuisance parameter. This property also simplifies the design of test statistics for different sources of irreplicability: nearly all existing test statistics can be easily modified and incorporated within our model. In our numerical examples, we illustrate how users can tailor test statistics to capture various factors contributing to irreplicable signals. A workflow of DiscRep is shown in Fig. 1.

### 2.4 Posterior-PRP

Formally, the posterior-PRP is defined as


(6) $$p_{\text{posterior-PRP}}:=\mathbb{P}\left(T\left({\hat{\beta }}^{\prime },\theta \right)\geq T\left(\hat{\beta },\theta \right)|\hat{\beta },R_{0}\right)=\iint_{\left(\theta ,{\hat{\beta }}^{\prime }\right)}1\left(T\left({\hat{\beta }}^{\prime },\theta \right)\geq T\left(\hat{\beta },\theta \right)\right)\times \left(\prod_{i=1}^{m}p\left({\hat{\beta }}_{i}^{\prime }|\theta \right)\right)p\left(\theta |\hat{\beta }\right)d\theta d{\hat{\beta }}^{\prime },$$

where $\theta =(\bar{\beta },k)$, $\hat{\beta }=({\hat{\beta }}_{1},\ldots ,{\hat{\beta }}_{m})$ and ${\hat{\beta }}^{\prime }=({\hat{\beta }}_{1}^{\prime },\ldots ,{\hat{\beta }}_{m}^{\prime })$ are observed data and replicated data under our generative model, respectively. $T(\hat{\beta },\theta )$ is a test quantity that measures the discrepancy between data and model where $R_{0}$ means that the probability is evaluated under the reference replicability model with respect to θ and ${\hat{\beta }}^{\prime }$. However, in practice, this posterior-PRP is often not directly computable due to the complexity of the involved distributions and integrals. Therefore, we propose a sampling scheme to obtain the posterior-PRP. Algorithm 1 outlines this method, using the Metropolis–Hastings (MH) algorithm (Metropolis et al. 1953, Hastings, 1970). Detailed implementation of Algorithm 1 is provided in Algorithm S1 in Section C.4 of the Supplementary Notes, available as supplementary data at *Bioinformatics* online.

Algorithm 1: Approximating posterior-PRP by posterior sampling

**Input:** total iterations *N*, observed effects ${\hat{\beta }}_{j}$ and variances $\hat{\sigma }j{\;}^{2}$, j=1,…,m.

**Output:**  $p_{\text{posterior-PRP}}$.

**Procedure:** 

 Initialize count←0

 **for each iteration in** *N* **do** 

  (a) Sample $\left(\bar{\beta },k\right)$ using the Metropolis–Hastings algorithm.

  (b) For each j=1,…,m, sample $\beta_{j}$ from normal distribution

   with {mean $\frac{{\hat{\sigma }}_{j}^{2}\bar{\beta }+k^{2}{\bar{\beta }}^{2}{\hat{\beta }}_{j}}{{\hat{\sigma }}_{j}^{2}+k^{2}{\bar{\beta }}^{2}}$ and variance $\frac{k^{2}{\bar{\beta }}^{2}{\hat{\sigma }}_{j}^{2}}{{\hat{\sigma }}_{j}^{2}+k^{2}{\bar{\beta }}^{2}}$.}

  (c) For each j=1,…,m, sample ${\hat{\beta }}_{j}\prime ∼N(\beta_{j},{\hat{\sigma }}_{j}^{2})$.

  (d) Compute test statistics $T(\hat{\beta }\prime ,(\bar{\beta },k))$ and $T(\hat{\beta },(\bar{\beta },k))$.

  (e) **if**  $T(\hat{\beta }\prime ,(\bar{\beta },k))\geq T(\hat{\beta },(\bar{\beta },k))$  **then** 

    count←count+1.

    **end if** 

  **end for** 

  Compute one-sided *p*-value: $p_{\text{posterior-PRP}}=\frac{\text{count}}{N}$.

  **return**  $p_{\text{posterior-PRP}}$.

 **end procedure** 

### 2.5 Properties of posterior-PRP

While posterior-PRP under the distinguishability criterion is not directly computable, it admits a closed-form analytical solution under the fixed effect model (k=0), as presented in Section D of the Supplementary Notes, available as supplementary data at *Bioinformatics* online. We now have the model with *m* studies


$$\bar{\beta }∼1\;\;\text{and}\;\;{\hat{\beta }}_{j}|\bar{\beta }∼N(\bar{\beta },{\hat{\sigma }}_{j}^{2}),\;\text{for}\;j=1,\ldots ,m.$$

As in Supplementary Notes, available as supplementary data at *Bioinformatics* online, we give the explicit expression


(7) $$p_{\text{posterior-PRP}}(c_{0})=\int_{c_{0}}^{\infty }\left[1-F_{\chi_{m}^{2}}(t)\right]f_{\chi_{1}^{2}}(t-c_{0})dt,$$

where $F_{\chi_{m}^{2}}$ and $f_{\chi_{1}^{2}}$ are the CDF and PDF of the $\chi^{2}$ distribution with *m* and 1 degrees of freedom, respectively and $c_{0}$ is derived from the deviation of each study’s observed effect from the summary effect, weighted by the inverse of the study’s variance. Specifically,


$$c_{0}=\sum_{j=1}^{m}w_{j}{\left({\hat{\beta }}_{j}-\mu_{\bar{\beta }}\right)}^{2},\;w_{j}=\frac{1}{{\hat{\sigma }}_{j}^{2}}\;\text{and}\;\mu_{\bar{\beta }}=\frac{\sum_{j=1}^{m}w_{j}{\hat{\beta }}_{j}}{\sum_{j=1}^{m}w_{j}}.$$

Equation (7) reveals how Bayesian *P*-value behaves as a function of $c_{0}$. Posterior-PRP is a non-increasing function of $c_{0}$. This behavior aligns with our intuition: when the observed data diverges much from what is predicted by the model, the posterior-PRP should decrease, signaling a poorer fit. Conversely, when $c_{0}=0$, meaning the observed data perfectly fits the model, the posterior-PRP reaches its maximum value. This property ensures that the posterior-PRP provides a meaningful measure of fit between the model and the data. Small values of $p_{\text{posterior-PRP}}$ indicate significant departures from the model, while large values suggest that the data is consistent with the model.

We further derive a clear and concise upper bound for posterior-PRP in the fixed effect model, which is given by the regularized incomplete beta function $I_{\frac{1}{2}}\left(\frac{1}{2},\frac{m}{2}\right)$, where $I_{d_{1}t/(d_{1}t+d_{2})}\left(\frac{d_{1}}{2},\frac{d_{2}}{2}\right)=P(F_{d_{1},d_{2}}\leq t)$ and $F_{d_{1},d_{2}}$ denote *F*-distribution with $d_{1}$ and $d_{2}$ degrees of freedom. This result provides an elegant way to express the maximum value of the posterior-PRP. It reveals that as *m* grows, the upper bound of the posterior-PRP increases and converges toward 1. On the other hand, for small *m*, the upper bound provides a key insight into how posterior-PRPs differ from frequentist *P*-values. Specifically, the upper bounds for m=2,3,4 are approximately 0.7071,0.8183,0.8804 that are less than 1, as we compute in the Supplementary Notes, available as supplementary data at *Bioinformatics* online. Moreover, the mean of the posterior-PRPs across different values of *m* is always 0.5 under the null, as proven in Theorem 1 by Meng (1994). In frequentist settings, *P*-values are typically uniformly distributed with mean 0.5 and upper bound 1 under the null hypothesis.

Although these properties are derived within the fixed effect model, they are not confined to this context. The same behavior of posterior-PRPs with respect to $c_{0}$ and *m* can be expected within the framework of the distinguishability criterion as well. The distinguishability criterion, which evaluates replicability by quantifying heterogeneity among replicable effects, still relies on Bayesian *P*-values to assess model fit, regardless of the model’s complexity. Detailed derivations of properties can be found in Section D of the Supplementary Notes, available as supplementary data at *Bioinformatics* online. We also present the upper bound of posterior-PRP, the relationship between posterior-PRP and $c_{0}$ in Fig. 5, available as supplementary data at *Bioinformatics* online, and the histogram of posterior-PRP for different values of *m* in Fig. 6, available as supplementary data at *Bioinformatics* online.

## 3 Results

### 3.1 Simulation studies

#### 3.1.1 Batch effect detection

We perform simulation studies to evaluate the effectiveness of our proposed computational methods in detecting batch effects, a prevalent source of irreplicability in high-throughput biological experiments. We use a linear model framework to simulate the impact of batch effects in a case-control study. We simulate 10 experiments (m=10), with four experiments having batch effects and six experiments not having batch effects. We design the simulation with 100 individuals per experiment, targeting a quantitative trait. Binary batch labels ($c_{j},j=1,\ldots ,m$) are generated from the case-control labels ($x_{j},j=1,\ldots ,m$). Specifically, approximately 80% of the labels $c_{j}=x_{j}$ and the remaining labels $c_{j}=1-x_{j}.$ The case-control labels ($x_{j}$) follow a Bernoulli distribution with probability of success 0.4.

For experiments with batch effects, the outcome variable is modeled using the linear regression as follows


(8) $$y_{j}=\beta_{0}+\beta_{\text{true}}x_{j}+\beta_{\text{batch}}c_{j}+\epsilon_{j},\;\epsilon_{j}∼N(0,1),$$

where $y_{j},j=1,\ldots ,m$ is a continuous trait, $\beta_{0}$ is the intercept, $\beta_{\text{true}}$ is the regression coefficient of case-control status and $\beta_{\text{batch}}$ is the regression coefficient of the batch effect. For experiments without batch effects, we remove $\beta_{\text{batch}}c_{j},j=1,\ldots ,m,$ from Equation (8).

The true case-control association $\beta_{\text{true}}$ is sampled from $N(0.1,k^{2}0.1^{2})$, where *k* is 0.25 reflecting a low-level heterogeneity. The regression coefficient of the batch effects $\beta_{\text{batch}}$ is sampled from a normal distribution $N(0,\eta^{2}),$ where η varies from 0 to 0.8 (from weak to strong) to represent different levels of variability (batch contamination levels). A simple linear model without batch effect is fitted to each experiment. The estimated regression coefficient of case-control status and its standard error are used as observed data (${\hat{\beta }}_{j},{\hat{\sigma }}_{j}$, j=1,…,m) in the posterior predictive checking model. This simulation is repeated 2000 times for each value of η. The total number of Markov Chain Monte Carlo (MCMC) iterations *N* is 10 ,000with a burn-in rate r of 0.05. Additionally, we compare Bayesian *P*-values with their frequentist counterpart, derived from Cochran’s *Q* statistic (Cochran 1950, Patil 1975)


(9) $$Q=\sum_{j=1}^{m}w_{j}{\left({\hat{\beta }}_{j}-\frac{\sum_{j=1}^{m}w_{j}{\hat{\beta }}_{j}}{\sum_{j=1}^{m}w_{j}}\right)}^{2},$$

where $w_{j}=\frac{1}{{\hat{\sigma }}_{j}^{2}}$. Cochran’s *Q* statistic assumes a fixed effect model and follows a $\chi^{2}$ distribution with m−1 degrees of freedom. The test quantity we use to detect batch effects is the modified *Q* statistic as follows:


(10) $$T(\hat{\beta },(\bar{\beta },k))=\sum_{j=1}^{m}{\tilde{w}}_{j}{\left({\hat{\beta }}_{j}-\bar{\beta }\right)}^{2},$$

where ${\tilde{w}}_{j}={\left({\hat{\sigma }}_{j}^{2}+k^{2}{\bar{\beta }}^{2}\right)}^{-1}$, and $\bar{\beta }$ and *k* are estimated according to the reference replicability model using Algorithm 1, available as supplementary data at *Bioinformatics* online. We flag datasets with posterior-PRPs below the conventional significance threshold of 0.05.

**Figure 2:**
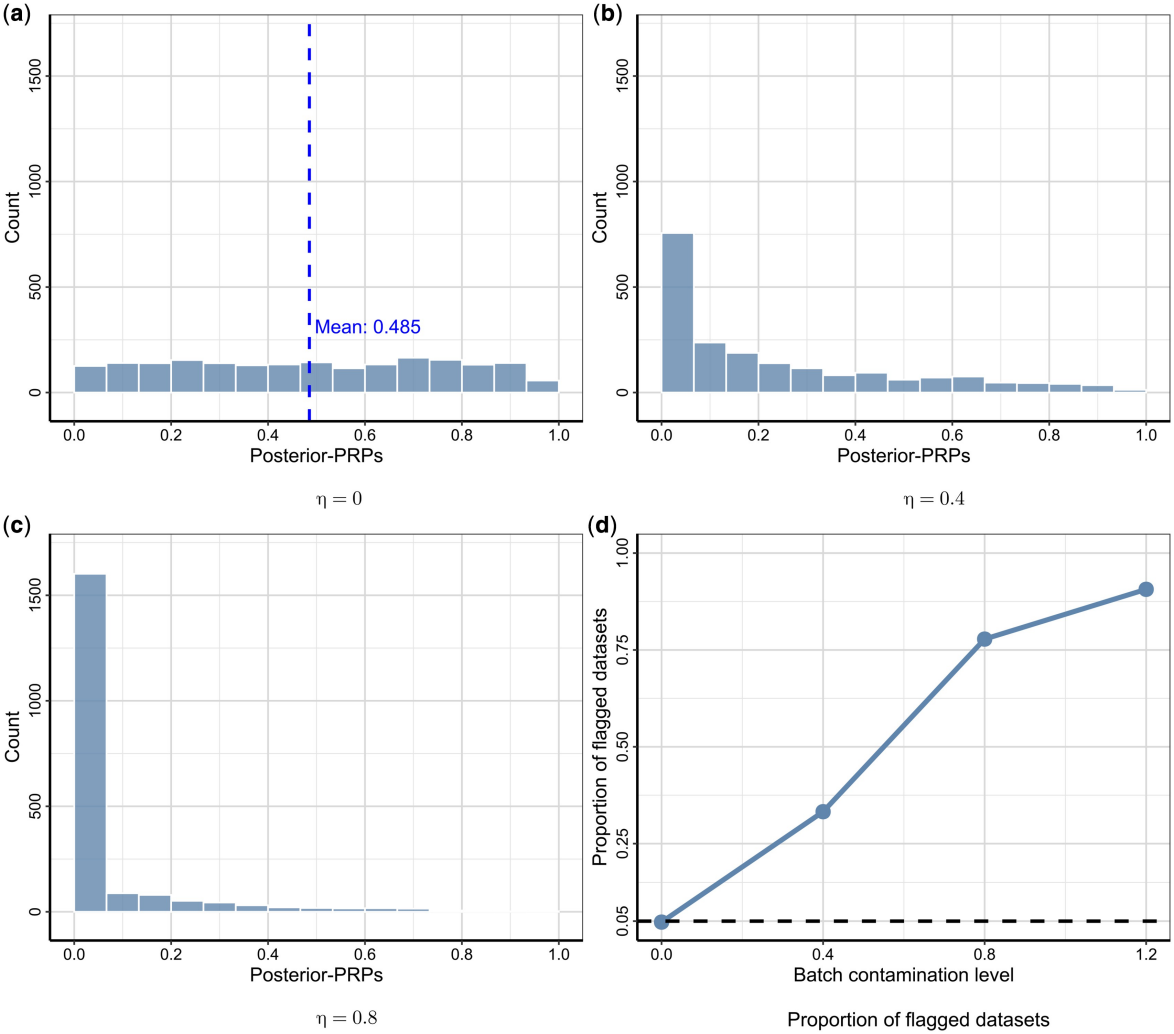
Batch effect detection. This figure presents the empirical distributions of posterior-PRPs for different batch contamination levels, η. The panels illustrate the distribution of posterior-PRPs across 2000 simulations for each of three distinct batch effect levels: (a) η=0 (no batch effect), (b) η=0.4 (moderate batch effect), and (c) η=0.8 (strong batch effect). Panel (d) illustrates the proportion of flagged experiments of posterior-PRPs to batch effects as a function of η, demonstrating the growing capability of posterior-PRPs to detect batch effects with increasing levels of contamination.

Figure 2a illustrates the behavior of posterior-PRPs under a contamination-free scenario (i.e. the reference replicability model), where the average of observed *P*-values closely aligns with the theoretical expectation of 0.5 (Meng 1994). Despite conceptual differences, Bayesian *P*-values exhibit distributions similar to frequentist *P*-values derived from Cochran’s *Q* tests (Figs 1 and 2, available as supplementary data at *Bioinformatics* online) while allowing heterogeneity among replicable results. Figure 2b and c shows Bayesian *P*-value distributions in the presence of irreproducible signals. The strong positive skewness, characterized by an excess of small *P*-values, indicates a poor fit of the reference replicability model, because the observed variability exceeds the level of expected heterogeneity. Additionally, we observe increasing skewness in posterior-PRP distributions as batch effect magnitude grows. Finally, we compute the proportion of flagged datasets at each level of batch contamination. Figure 2d demonstrates that our method is well-calibrated under the reference replicability model and effectively detects unobserved batch effects.

**Figure 3:**
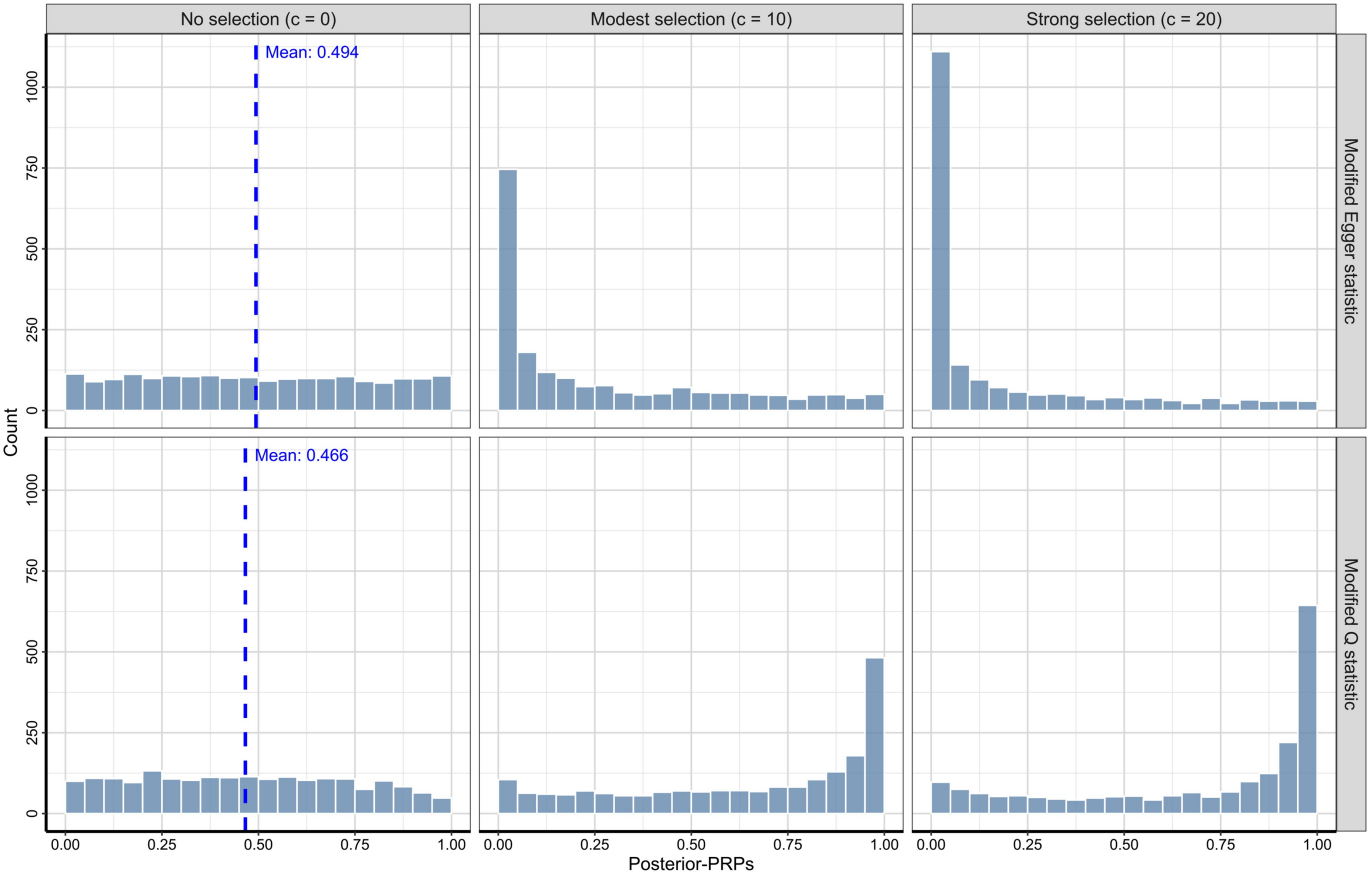
Publication bias detection across varying selection strengths. Each panel shows the distribution of posterior-PRPs derived from modified *Q* and Egger statistics under different selection conditions: no selection (c=0), modest selection (c=10), and strong selection (c=20).

We conduct MCMC convergence diagnostics to evaluate the sampling performance. Figure 3, available as supplementary data at *Bioinformatics* online, presents the trace plots and autocorrelation function (ACF) curves for four randomly selected simulations (distinguished by color). The trace plots demonstrate stable post-burn-in trajectories with no systematic drift across η∈{0,0.4,0.8}. The ACF curves decline rapidly toward zero over lags, indicating limited long-range dependence in the retained draws. Mean effective sample sizes (ESS) remain high for all settings (ESS=2897.7,2897.1,2724.8 for η=0,0.4,0.8, respectively). Mean acceptance ratios are moderate and stable (0.3837,0.3841,0.3722 for η=0,0.4,0.8, respectively). The mean of sampled $\bar{\beta }$ over 2000 simulations is 0.1 at η=0, 0.1035 at η=0.4, and 0.1135 at η=0.8 (true $\bar{\beta }=0.1$).

#### 3.1.2 Publication bias detection

Next, we conduct simulations to assess the effectiveness of the proposed methods in detecting publication bias, a key factor contributing to irreplicable results in practice. Publication bias often leads to the selective reporting of significant results, thus skewing the overall interpretation of research findings. We consider a dataset with 20 studies of varied sample sizes: 10 with 100 samples, six with 200 samples, and four with 240 samples. The case-control study comprises 60% control group samples ($x_{i}=0$) and 40% treatment group samples ($x_{i}=1$). Without loss of generality, we use *i* to denote a generic sample, and *j* to denote a generic study. The binary outcome $y_{i}$ is generated from the following model:


(11) $$\mathrm{logit}[P(y_{i}=1)]=\text{log}\;\left[\frac{P(y_{i}=1)}{1-P(y_{i}=1)}\right]=\beta_{0}+\beta_{\text{true}}x_{i},$$

where $\beta_{0}$ is the intercept and $\beta_{\text{true}}$ is the log odds ratio of the treatment and control groups. For the *j*th study, generate $u_{j}$ from a Uniform[0.6,0.9] distribution. We generate $\beta_{0}$ from $\text{log}\;\frac{u_{j}}{1-u_{j}}$ and $\beta_{\text{true}}$ from $N(\text{log}(\frac{2}{3}),k^{2}{\;\text{log}\;}^{2}(\frac{2}{3}))$, where *k* is 0.25 incorporating a low-level heterogeneity. The outcome $y_{i}$ is generated from a Bernoulli distribution with probability of success $e^{\beta_{0}}/(1+e^{\beta_{0}})$ and $e^{\beta_{0}+\beta_{\text{true}}}/(1+e^{\beta_{0}+\beta_{\text{true}}})$ for the control group and the case group, respectively. Summary statistics, including estimated log odds ratios (${\hat{\beta }}_{j}$), standard errors (${\hat{\sigma }}_{j}$), and *P*-values (*P_j_*), are obtained by fitting data with (11). To introduce publication bias into the simulated datasets, we implement a selection process. In this process, less significant results tend to be discarded and regenerated with higher probability determined by a parameter *c* characterizing the strength of the selection. Specifically, for each study, we use a Bernoulli distribution with probability of success $e^{-cp_{j}{\;}^{3/2}}$ to decide whether to keep the study in the analysis (Macaskill et al. 2001). If the Bernoulli random variable generates a value of 1, the study is kept, otherwise, it is excluded. We stop generating data once all 20 studies are collected. We simulate 2000 datasets to explore different selection strengths with c=0 (no selection), c=10 (modest selection), and c=20 (strong selection).

We implement modified Egger’s statistic, designed to test the asymmetry of funnel plots (Egger et al. 1997), to calculate our posterior-PRP and to assess the potential publication bias. For comparison, we also compute posterior-PRP values using the modified Cochran’s *Q* statistic (10). We obtain the modified Egger statistic by fitting the following regression model and using its standardized intercept estimate as the test statistic. The regression model is


$$\frac{{\hat{\beta }}_{j}}{\sqrt{{\hat{\sigma }}_{j}^{2}+k^{2}{\bar{\beta }}^{2}}}=\alpha_{0}+\alpha_{1}\frac{1}{\sqrt{{\hat{\sigma }}_{j}^{2}+k^{2}{\bar{\beta }}^{2}}}+\epsilon_{j},\;\epsilon_{j}∼N(0,\sigma_{\epsilon }^{2}),$$

where j=1,…,m, $\alpha_{0}$ is the intercept, $\alpha_{1}$ is the slope, $\sigma_{\epsilon }^{2}$ is the variance of the error term, and ($\bar{\beta },k$) can be obtained under the reference replicability model using Algorithm 1, available as supplementary data at *Bioinformatics* online. The modified Egger test evaluates the null hypothesis of no publication bias: $\alpha_{0}=0$. In the absence of publication bias, $\alpha_{0}$ should be zero, indicating a symmetrical distribution of study results around the true effect. A significant test result suggests the presence of publication bias. When publication bias exists, smaller studies with non-significant findings are often unpublished, leading to an asymmetrical distribution. This asymmetry causes the intercept to deviate from zero. Specifically, our test quantity $T_{E}(\hat{\beta },(\bar{\beta },k))$ designed for the publication bias is the squared estimation of $\alpha_{0}$ divided by corresponding variance as follows. Let $r={\left(r_{1},\ldots ,r_{m}\right)}^{T}={\left(\frac{{\hat{\beta }}_{1}}{\sqrt{{\hat{\sigma }}_{1}^{2}+k^{2}{\bar{\beta }}^{2}}},\ldots ,\frac{{\hat{\beta }}_{m}}{\sqrt{{\hat{\sigma }}_{m}^{2}+k^{2}{\bar{\beta }}^{2}}}\right)}^{T}\in R^{m}$ and $d={\left(d_{1},\ldots ,d_{m}\right)}^{T}={\left(\frac{1}{\sqrt{{\hat{\sigma }}_{1}^{2}+k^{2}{\bar{\beta }}^{2}}},\ldots ,\frac{1}{\sqrt{{\hat{\sigma }}_{m}^{2}+k^{2}{\bar{\beta }}^{2}}}\right)}^{T}\in R^{m}$. Based on the ordinary least squares, we get the estimations of $\alpha_{1}$, $\alpha_{0}$, $\sigma_{\epsilon }^{2}$, and variance estimation of $\alpha_{0}$ by


$$\begin{array}{c} {\hat{\alpha }}_{1}=\frac{\sum_{j=1}^{m}\left(d_{j}-\bar{d}\right)(r_{j}-\bar{r})}{\sum_{j=1}^{m}{\left(d_{j}-\bar{d}\right)}^{2}},\;{\hat{\alpha }}_{0}=\bar{r}-{\hat{\alpha }}_{1}\bar{d},\\ {\hat{\sigma }}_{\epsilon }^{2}=\frac{1}{m-2}∥r-{\hat{\alpha }}_{0}1_{m}-{\hat{\alpha }}_{1}d{∥}_{2}^{2},\;1_{m}={\left(1,\ldots ,1\right)}^{T}\in R^{m},\\ {\hat{\sigma }}_{\alpha_{0}}^{2}={\hat{\sigma }}_{\epsilon }^{2}\left(\frac{1}{m}+\frac{{\bar{d}}^{2}}{\sum_{j=1}^{m}{\left(d_{j}-\bar{d}\right)}^{2}}\right), \end{array}$$

where $\bar{d}$ and $\bar{r}$ are the mean of d and r, respectively. The test quantity designed for the publication bias is


$$T_{E}(\hat{\beta },(\bar{\beta },k))=\frac{{\hat{\alpha }}_{0}^{2}}{{\hat{\sigma }}_{\alpha_{0}}^{2}}.$$

Figure 3 illustrates the impact of selection strength on the sensitivity of the modified Egger and *Q* tests to detect publication bias. As the selection strength increases (*c* values), the Egger-based test becomes more powerful in identifying publication bias, as indicated by a higher frequency of small *P-*values. In contrast, tests based on *Q*-values appear less effective in detecting irreplicability due to publication bias. This observation suggests that no single test or statistic can capture all sources of replicability, emphasizing the need to employ multiple test statistics in our proposed computational framework for a comprehensive examination of various aspects of the observed data.

### 3.2 Case studies

#### 3.2.1 Assessing eQTL tissue specificity

Meta-analysis techniques are essential in combining eQTL results from multiple tissues to increase the power of detection and to evaluate the consistency of genetic effects across tissues. This is particularly important given the tissue-specific nature of gene expression and regulation. The challenge lies in distinguishing true biological cross-tissue variability from noise. The NIH Genotype-Tissue Expression (GTEx) project provides genotype and expression data across different tissues, which we use to identify tissue-specific eQTLs. Our analysis focuses on the replicability of genetic associations between variants and the expression levels of target genes across multiple tissues, where the reference null model is set up to describe consistent eQTL effects across all tissues. Although our method can scale to simultaneously analyze all 49 GTEx tissues, for illustration and comparison with existing multi-tissue eQTL analysis method, we restrict our analysis to three tissues: artery aorta, liver, and skeletal muscle, with sample sizes of 387, 208, and 706 respectively. These three tissues are selected because their RNA-seq experiments are of relatively high quality. The data are processed to ensure quality and no selection bias, and we implement our method to assess the replicability of eQTL findings. Finally, we obtain summary association statistics as inputs for 18 826 gene-SNP pairs across these three tissues from the GTEx portal.

**Figure 4:**
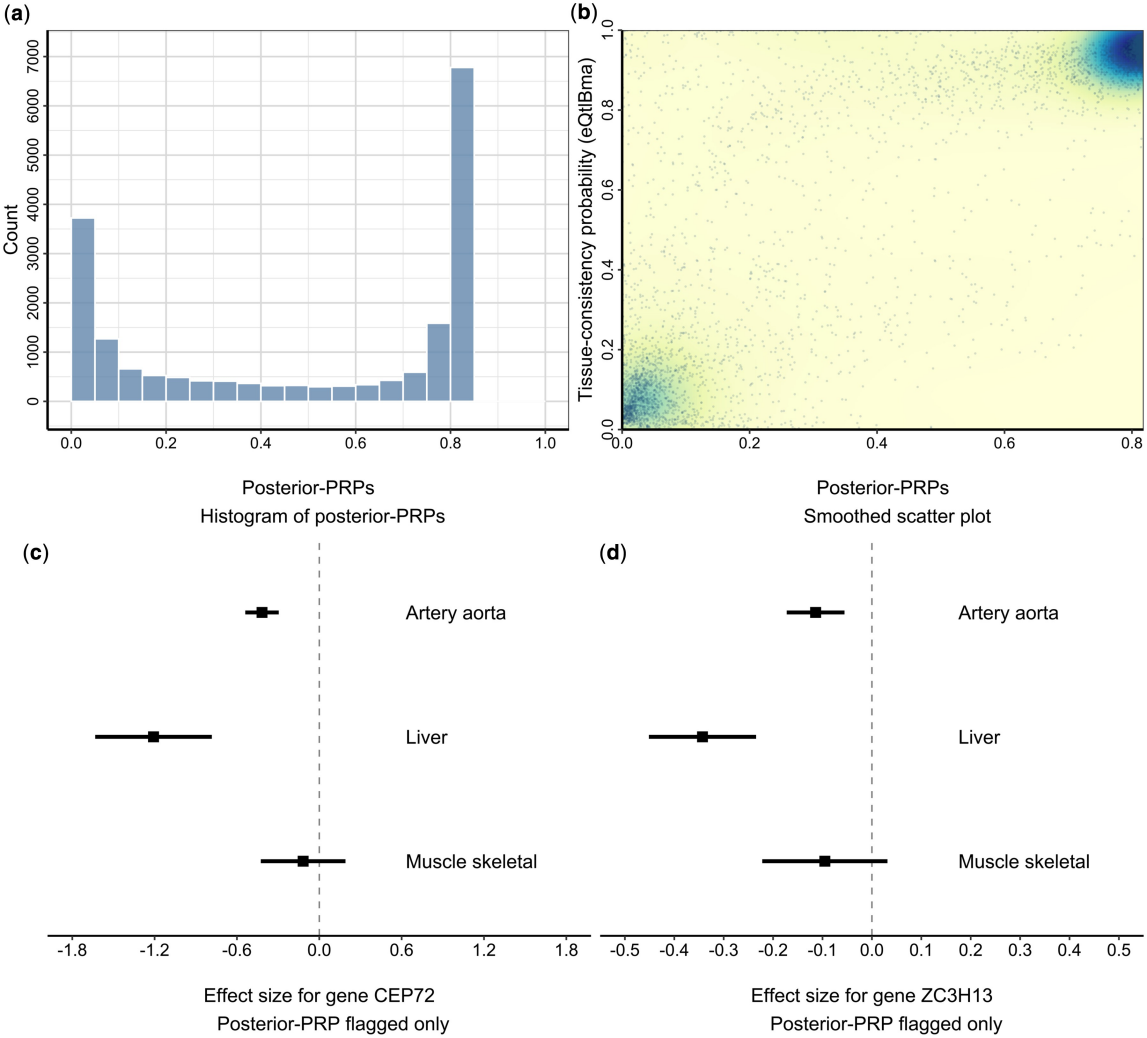
eQTL analysis across multiple tissues. These figures summarize cross-tissue eQTL effect patterns across three GTEx tissues (artery aorta, liver, and skeletal muscle) for 18 826 gene–SNP pairs, comparing posterior-PRPs with tissue-consistency probabilities from eQtlBma. (a) Histogram of posterior-PRPs; small values are interpreted here as cross-tissue heterogeneity/tissue specificity. (Note that the maximum posterior-PRP value is strictly less than 1, as explained in Section 2.5). (b) A smoothed version of a scatter plot between posterior-PRPs and eQtlBma. Areas with greater point density indicates more scatter clustering. (c, d) The forest plots visualize the effect sizes and their corresponding confidence intervals for *CEP72* and *ZC3H13* genes flagged by posterior-PRP but not by eQtlBma. Both genes exhibit directionally consistent but quantitatively different effects across tissues. The horizontal axis represents the zero effect size.

We compare our method, which implements posterior-PRP using the modified Cochran’s *Q*  (10), with the eQtlBma method (Flutre et al. 2013, Wen and Stephens 2014). Given the small sample size of the GTEx data, we assume that there is at most one eQTL within the cis region of each target gene. This simplified assumption, also adopted by eQtlBma, enables the computation of a gene-level eQTL effect and its corresponding standard error via an expectation–maximization algorithm for each target gene in a given tissue. These estimates of the effects at the gene level then serve as input for computing the posterior-PRPs of the 18 826 genes in the three tissues (Fig. 4a). In this application, we report small posterior-PRPs as evidence of tissue specificity, rather than labeling them as “irreplicable” effects. We compare our method with eQtlBma because both methods focus on addressing the heterogeneity issue in cross-tissue analysis. While eQtlBma uses a Bayesian model to combine eQTL results from multiple tissues, our approach introduces a distinguishability criterion, which provides a direct measure of tissue-specific heterogeneity through misclassification probability. To summarize the overall fraction of cross-tissue consistent eQTLs, we can use the posterior-PRPs under the mixture model framework (Efron et al. 2001, Wen 2017, Cao et al. 2022). The expected value of a posterior-PRP under this model can be expressed as:


$$\begin{array}{cc} E(\text{posterior-PRP})=\pi_{r}\times \frac{1}{2}+(1-\pi_{r})\times & \\ E(\text{posterior-PRP}|\text{tissue-specific}\;\text{scenario}), & \end{array}$$

where $\pi_{r}$ represents the probability that a gene–SNP pair has a cross-tissue consistent (shared) eQTL effect, $\frac{1}{2}$ is the expected posterior-PRP under the reference shared-effect model $R_{0}$ (Meng 1994), and E(posterior-PRP∣tissue-specificscenario) is the expected posterior-PRP under the tissue-specific scenario. We have $\pi_{r}\times \frac{1}{2}\leq E(\text{posterior-PRP})$, since E(posterior-PRP∣tissue-specificscenario)≥0. Based on this, an empirical upper bound on $\pi_{r}$ can be obtained by the formula ${\hat{\pi }}_{r}=2\times \mathrm{mean}(\text{posterior-PRP}),$ where mean(posterior-PRP) is the sample mean of posterior-PRPs.

Based on the empirical distribution of posterior-PRP values and a simple mixture model, we estimate an upper bound (i.e. a potential overestimation) of 0.972 for tissue-consistent eQTLs across the three tissues, suggesting that the majority of detected eQTLs are tissue-consistent. This finding is quantitatively consistent with previous reports in the literature (Flutre et al. 2013). Nevertheless, we also observe an excess of small Bayesian *P*-values in Fig. 4a: 19.7% of the 18 826 genes have posterior-PRP values ≤0.05, indicating that tissue-specific eQTL effects are not uncommon.

Compared to the posterior probabilities of consistent eQTLs estimated by eQtlBma, as a method specifically designed to evaluate tissue-consistency of eQTLs, we find that the posterior-PRPs from our method are highly correlated with the eQtlBma tissue-consistency probabilities (Spearman’s correlation = 0.712; Fig. 4b). In addition to methodological differences between the two approaches, we note that tissue specificity is defined somewhat differently in eQtlBma, which emphasizes identifying qualitatively distinct association patterns (i.e. presence versus absence of an association). In contrast, our proposed method also accounts for quantitative differences in association strength (i.e. strong versus weak associations in the same direction). Therefore, the imperfect correlation between the two sets of results is expected.

Our investigations of specific genes with seemingly discordant conclusions from posterior-PRP and eQtlBma further support this interpretation (Fig. 4). Figure 4c and d shows forest plots for two such protein-coding genes, *CEP72* and *ZC3H13*, with posterior-PRPs <0.05 but probability of a consistent configuration from eQtlBma >0.7. It is evident that in both cases the estimated effects are qualitatively consistent but quantitatively distinct among the three tissues. Notably, both *CEP72* and *ZC3H13* have been implicated in liver-specific functions and diseases. *CEP72* has been proposed as a potential biomarker for liver cancer, with associations to tumor development, stem cell-like properties, and metastasis (Ye et al. 2020). *ZC3H13* is downregulated in hepatocellular carcinoma (HCC), and low expression is linked to poor prognosis. Its overexpression, on the other hand, suppresses the migration and invasion of HCC cells, suggesting a tumor-suppressive role in liver cancer (Wu et al. 2022).

#### 3.2.2 Systematic review of published meta-analysis results

We apply the proposed computational methods to perform systematic reviews of three published meta-analyses in medical research. The first, by Stead et al. (2012), focuses on the effectiveness of nicotine gum in assisting smoking cessation, analyzing 56 studies treating the log risk ratio as the effect size. The second meta-analysis, conducted by Hróbjartsson and Gøtzsche (2010), looks into the impact of placebo interventions across various clinical conditions, including 109 studies that use the standardized mean difference as the effect size. The third, presented by Liu and Latham (2009), investigates the effects of progressive resistance strength training in comparison to a control group, drawing from 33 studies with the standardized mean difference as the effect size.

We first compute the posterior-PRPs using the modified *Q* statistic to assess the overall heterogeneity within each meta-analysis (Table 1). The results indicate that the first two meta-analyses exhibit a higher level of heterogeneity than expected under the reference replicability model (assuming a maximum $P_{\text{mis}}=0.05$), suggesting that some reported estimates may not be fully replicable. These findings are consistent with traditional heterogeneity assessments based on the fixed effect model: the $I^{2}$ values (Higgins and Thompson 2002, Higgins et al. 2003) for the three analyses are 0.42,0.44, and 0.17, respectively, where $I^{2}$ is calculated using 100%×(Q−(m−1))/Q where *Q* is Cochran’s *Q* heterogeneity statistic defined in (9).

**Table 1:** Results for the three meta-analyses.^a^

	Posterior-PRP	
	Modified	Modified
	*Q* statistic	Egger statistic
Stead *et al.* (2012)	0.015	0.212
Hróbjartsson and Gøtzsche (2010)	$1.1\times 10^{-4}$	0.044
Liu and Latham (2009)	0.307	0.897

a This table summarizes the findings from the three meta-analyses, focusing on detecting heterogeneity and publication bias. The first column lists the studies by their respective authors. The remaining columns report the *P*-values from two statistical tests: posterior-PRP (*Q*-based), and posterior-PRP (Egger-based). The posterior-PRP (*Q*-based) assesses heterogeneity. The posterior-PRP (Egger-based) focuses on identifying publication bias.

To further investigate whether the observed heterogeneity can be attributed to publication bias, we calculate posterior-PRPs using the modified Egger statistic (Table 1). Only the second meta-analysis yields a Bayesian *P*-value <.05, indicating that publication bias may be a factor contributing to the excessive heterogeneity observed in Hróbjartsson and Gøtzsche (2010). This finding is also consistent with the visual inspection of the funnel plots (Fig. 4, available as supplementary data at *Bioinformatics* online).

Finally, we compare the posterior-PRP results with the frequentist *P*-values based on the fixed effect model. As expected, the results are qualitatively similar (Table 1, available as supplementary data at *Bioinformatics* online). The key advantage of the proposed methods is their ability to make a stronger case for potential irreplicable findings. For example, the level of heterogeneity in the results from Hróbjartsson and Gøtzsche (2010) exceeds the expectations for replicable results, even when a modest degree of heterogeneity is allowed.

## 4 Discussion

In this article, we introduce a novel computational framework for quantifying and assessing replicability across multiple experimental results. The key innovation is the development of a distinguishability criterion, which enables principled accounting for inherent heterogeneity among experiments on a universally interpretable probability scale. Building on this criterion, we implement a flexible hierarchical model (i.e. the reference replicability model) that allows practitioners to define context-specific characteristics of replicable results. Finally, we proposed to evaluate the goodness-of-fit of the user-specified reference replicability model to the observed data, using posterior-PRPs to identify potentially irreplicable results. Our numerical illustrations, including simulation and real data studies, demonstrate the broad applicability of the proposed computational method across a wide range of scientific applications.

We choose to implement the posterior-PRP as a Bayesian *P*-value due to its conceptual advantages and computational feasibility within our proposed reference replicability model. Many authors (Meng 1994, Gelman 2005, 2013) have argued that Bayesian *P*-values offer a more intuitive interpretation than their frequentist counterparts. Moreover, implementation based on a hierarchical reference replicability model is streamlined and straightforward, whereas computing a frequentist *P*-value can be considerably more challenging. However, the statistical differences between Bayesian and frequentist *P*-values may hinder users’ comprehension of the analysis results. For example, Bayesian *P*-values are *not* expected to be uniformly distributed on [0,1] under the reference replicability model. In particular, the specific form of the posterior-PRP may have an upper bound strictly less than 1. We summarize and discuss several relevant properties of posterior-PRPs in Section 2 to facilitate better understanding of their behavior under the reference replicability model.

We demonstrate that multiple distinct sources can lead to irreplicable results, each producing unique patterns in the observed data. As a result, it is difficult, if not impossible, to rely on a single statistical test to uncover all instances of irreplicability. To address this challenge, we propose deliberately designing specialized test statistics that target specific, known sources of irreplicability within our framework. This approach is shown to enhance the sensitivity of irreplicability detection and provide deeper insights into its underlying causes. For example, while the meta-analysis by Stead et al. (2012) reports moderate heterogeneity without evidence of publication bias, the meta-analysis by Hróbjartsson and Gøtzsche (2010) reveals both high heterogeneity and the presence of publication bias. In contrast, the analysis by Liu and Latham (2009) indicates minimal heterogeneity and no publication bias. Together, these findings highlight the complex interplay between heterogeneity and publication bias, underscoring the need to consider both factors in a comprehensive evaluation of replicability.

In the multi-tissue eQTL study, we demonstrate that our framework can be used to highlight biologically meaningful cross-tissue heterogeneity (i.e. tissue-specific regulation). In such multi-context applications, a small posterior-PRP is interpreted as evidence against a reference replicability model across contexts, rather than a failure to replicate within each context. This formulation, along with our proposed method, can be further generalized to analyze statistical interactions between a discrete variable—mimicking multiple experiments—and a quantitative measurement. Our multi-tissue eQTL analysis yields results that are qualitatively similar to those of state-of-the-art approaches specifically designed to identify tissue-specific eQTLs, with the added benefit of detecting eQTLs that exhibit consistent directional effects but varying effect sizes across tissues.

From a broader perspective, we emphasize that the proposed method is designed to ***detect*** effects that exhibit heterogeneity beyond a pre-specified threshold. However, interpreting the ***causes*** of such inconsistency necessarily requires caution and careful, context-dependent consideration. For example, in a meta-analysis setting, a high level of heterogeneity in the effects of interest is typically unexpected, and the detection of inconsistent effects may point to study-level biases or other adversarial factors. In contrast, in our multi-tissue eQTL analysis, the data have been carefully examined and processed to mitigate potential technical confounders, such as batch effects. In this context, only when such artifacts have been reasonably ruled out, genuine biological mechanisms then become a more plausible explanation for the observed excessive heterogeneity.

## Supplementary Material

btag140_Supplementary_Data

## Data Availability

All processed data for simulations and real data analysis are available at https://github.com/PengWang96/DiscRep/tree/master/paper_repro. An archived snapshot of the code used in this article is available on Zenodo: https://doi.org/10.5281/zenodo.18672916. Three meta-analyses data of publication bias are available at https://www.cochranelibrary.com/cdsr/doi/10.1002/14651858.CD003974.pub3/full, https://www.cochranelibrary.com/cdsr/doi/10.1002/14651858.CD002759.pub2/full, and https://www.cochranelibrary.com/cdsr/doi/10.1002/14651858.CD000146.pub4/full. eQTL data are available at https://gtexportal.org/home/datasets.

## References

[btag140-B1] Begg CB , Mazumdar M. Operating characteristics of a rank correlation test for publication bias. Biometrics 1994;50:1088–101. 7786990

[btag140-B2] Begley CG , Ellis LM. Raise standards for preclinical cancer research. Nature 2012;483:531–3. 22460880 10.1038/483531a

[btag140-B3] Bogomolov M , Heller R. Replicability across multiple studies. Statist Sci 2023;38:602–20.

[btag140-B4] Cao H , Chen J, Zhang X. Optimal false discovery rate control for large scale multiple testing with auxiliary information. Ann Stat 2022;50:807–57. 37138896 10.1214/21-aos2128 PMC10153594

[btag140-B5] Cochran WG. The comparison of percentages in matched samples. Biometrika 1950;37:256–66. 14801052

[btag140-B6] Dekkers OM. Meta‐analysis: key features, potentials and misunderstandings. Res Pract Thromb Haemost 2018;2:658–63. 30349883 10.1002/rth2.12153 PMC6178740

[btag140-B7] Efron B , Tibshirani R, Storey JD et al Empirical bayes analysis of a microarray experiment. J Am Stat Assoc 2001;96:1151–60.

[btag140-B8] Egger M , Smith GD, Schneider M et al Bias in meta-analysis detected by a simple, graphical test. BMJ 1997;315:629–34. 9310563 10.1136/bmj.315.7109.629 PMC2127453

[btag140-B9] Flutre T , Wen X, Pritchard J et al A statistical framework for joint eQTL analysis in multiple tissues. PLoS Genet 2013;9:e1003486. 23671422 10.1371/journal.pgen.1003486 PMC3649995

[btag140-B10] Gelman A. Comment: fuzzy and Bayesian *p*-values and *u*-values. Statist Sci 2005;20:380–1.

[btag140-B11] Gelman A. Two simple examples for understanding posterior *p*-values whose distributions are far from uniform. Electron J Stat 2013;7:2595–602.

[btag140-B12] Gelman A , Meng X-L, Stern H. Posterior predictive assessment of model fitness via realized discrepancies. Stat Sin 1996;6:733–60.

[btag140-B13] Gelman A , Carlin JB, Stern HS et al Bayesian Data Analysis. New York, NY, USA: CRC Press, 2013.

[btag140-B14] Goodman SN , Fanelli D, Ioannidis JPA. What does research reproducibility mean? Sci Transl Med 2016;8:341ps12. 10.1126/scitranslmed.aaf5027 27252173

[btag140-B15] Hastings WK. Monte Carlo sampling methods using Markov chains and their applications. Biometrika 1970;57:97–109.

[btag140-B16] Higgins JP. Commentary: heterogeneity in meta-analysis should be expected and appropriately quantified. Int J Epidemiol 2008;37:1158–60. 18832388 10.1093/ije/dyn204

[btag140-B17] Higgins JP , Thompson SG. Quantifying heterogeneity in a meta-analysis. Stat Med 2002;21:1539–58. 12111919 10.1002/sim.1186

[btag140-B18] Higgins JP , Thompson SG, Deeks JJ et al Measuring inconsistency in meta-analyses. BMJ 2003;327:557–60. 12958120 10.1136/bmj.327.7414.557 PMC192859

[btag140-B19] Hróbjartsson A , Gøtzsche PC. Placebo interventions for all clinical conditions. Cochrane Database Syst Rev 2010;2010:CD003974. 15266510 10.1002/14651858.CD003974.pub2

[btag140-B20] IntHout J , Ioannidis JP, Rovers MM et al Plea for routinely presenting prediction intervals in meta-analysis. BMJ Open 2016;6:e010247. 10.1136/bmjopen-2015-010247 PMC4947751 27406637

[btag140-B21] Johnson WE , Li C, Rabinovic A. Adjusting batch effects in microarray expression data using empirical bayes methods. Biostatistics 2007;8:118–27. 16632515 10.1093/biostatistics/kxj037

[btag140-B22] Leek JT , Scharpf RB, Bravo HC et al Tackling the widespread and critical impact of batch effects in high-throughput data. Nat Rev Genet 2010;11:733–9. 20838408 10.1038/nrg2825 PMC3880143

[btag140-B23] Li Q , Brown JB, Huang H et al Measuring reproducibility of high-throughput experiments. Ann Appl Stat 2011;5:1752–79.

[btag140-B24] Li S , Sesia M, Romano Y et al Searching for robust associations with a multi-environment knockoff filter. Biometrika 2022;109:611–29. 38633763 10.1093/biomet/asab055 PMC11022501

[btag140-B25] Li Y , Zhou X, Chen R et al STAREG: statistical replicability analysis of high throughput experiments with applications to spatial transcriptomic studies. PLoS Genet 2024;20:e1011423. 39361716 10.1371/journal.pgen.1011423 PMC11478871

[btag140-B26] Lin L , Chu H. Quantifying publication bias in meta-analysis. Biometrics 2018;74:785–94. 29141096 10.1111/biom.12817 PMC5953768

[btag140-B27] Liu CJ , Latham NK. Progressive resistance strength training for improving physical function in older adults. Cochrane Database Syst Rev 2009;2009:CD002759. 19588334 10.1002/14651858.CD002759.pub2 PMC4324332

[btag140-B28] Macaskill P , Walter SD, Irwig L. A comparison of methods to detect publication bias in meta-analysis. Stat Med 2001;20:641–54. 11223905 10.1002/sim.698

[btag140-B29] McKenzie JE , Veroniki AA. A brief note on the random-effects meta-analysis model and its relationship to other models. J Clin Epidemiol 2024;174:111492. 39098563 10.1016/j.jclinepi.2024.111492

[btag140-B30] Meng X-L. Posterior predictive *p*-values. Ann Stat 1994;22:1142–60.

[btag140-B31] Metropolis N , Rosenbluth AW, Rosenbluth MN et al Equation of state calculations by fast computing machines. J Chem Phys 1953;21:1087–92. 10.1063/5.0309018 41342507

[btag140-B32] Patil KD. Cochran’s *q* test: exact distribution. J Am Stat Assoc 1975;70:186–9.

[btag140-B33] Patil P , Peng RD, Leek JT. What should researchers expect when they replicate studies? A statistical view of replicability in psychological science. Perspect Psychol Sci 2016;11:539–44. 27474140 10.1177/1745691616646366 PMC4968573

[btag140-B34] Rubin DB. Bayesianly justifiable and relevant frequency calculations for the applied statistician. Ann Statist 1984;12:1151–72.

[btag140-B35] Seymour G. Predictive Inference. New York, NY, USA: CRC Press, 1993.

[btag140-B36] Stead LF , Perera R, Bullen C et al Nicotine replacement therapy for smoking cessation. Cochrane Database Syst Rev 2012;11:CD000146. 18253970 10.1002/14651858.CD000146.pub3

[btag140-B37] Wen X. Bayesian model selection in complex linear systems, as illustrated in genetic association studies. Biometrics 2014;70:73–83. 24350677 10.1111/biom.12112 PMC3954315

[btag140-B38] Wen X. Robust Bayesian FDR control using bayes factors, with applications to multi-tissue eQTL discovery. Stat Biosci 2017;9:28–49.

[btag140-B39] Wen X , Stephens M. Bayesian methods for genetic association analysis with heterogeneous subgroups: from meta-analyses to gene–environment interactions. Ann Appl Stat 2014;8:176–203. 26413181 10.1214/13-AOAS695 PMC4583155

[btag140-B40] Wu S , He G, Liu S et al Identification and validation of the N6-methyladenosine RNA methylation regulator ZC3H13 as a novel prognostic marker and potential target for hepatocellular carcinoma. Int J Med Sci 2022;19:618–30. 35582419 10.7150/ijms.69645 PMC9108408

[btag140-B41] Ye C , Zhang X, Chen X et al Multiple novel hepatocellular carcinoma signature genes are commonly controlled by the master pluripotency factor OCT4. Cell Oncol (Dordr) 2020;43:279–95. 31848930 10.1007/s13402-019-00487-3 PMC12990707

[btag140-B42] Zhao Y , Wen X. Statistical assessment of replicability via Bayesian model criticism. arXiv, arXiv:2105.03993, 2021, preprint: not peer reviewed.

[btag140-B43] Zhao Y , Sampson MG, Wen X. Quantify and control reproducibility in high-throughput experiments. Nat Methods 2020;17:1207–13. 33046893 10.1038/s41592-020-00978-4 PMC8240032

