# Supplementary material for: Diagnosing scientific replicability through probabilistic distinguishability
Source: Bioinformatics. 2026 Mar 23;42(5):btag140. doi: 10.1093/bioinformatics/btag140 (PMC13148965; doi:10.1093/bioinformatics/btag140)
Supplement: btag140_Supplementary_Data [file btag140_supplementary_data.pdf]

Supplementary Notes for "Diagnosing scientific replicability through probabilistic distinguishability"

Peng Wang[1,4], Hongyuan Cao[2,4*] and Xiaoquan Wen[3*]

The Supplementary Notes provide a detailed explanation of the methodologies and analyses supporting the main paper. Supplementary tables and figures in Section A and B are provided to support the results of the simulations and real data analyses. Section C includes a comprehensive analysis of the misclassification probability based on the distinguishability criterion and the details of the sampling methodology under the reference replicability model. Section D investigates the derivation and theoretical properties of posterior-predictive replication p-values (posterior-PRPs) within the fixed effect model.

[1]School of Mathematics, Jilin University, Changchun, Jilin 130012, China. [2]Department of Statistics, Florida State University, Tallahassee, FL 32306, U.S.A. [3]Department of Biostatistics, University of Michigan, Ann Arbor, MI 48109, U.S.A. [4]Department of Statistics and Data Science, Mohamed bin Zayed University of Artificial Intelligence, Abu Dhabi, United Arab Emirates.

[*]Co-corresponding authors: hcao@fsu.edu; xwen@umich.edu

A Supplementary Tables

		p-value				
	I^2(%)	Q test	posterior-PRP (Q-based)	posterior-PRP (Egger-based)	Egger	Begg
Stead et al. [2012]	42	0.002	0.015	0.212	0.174	0.136
Hróbjartsson and Gøtzsche [2010]	44	3.7×10^{-6}	1.1×10^{-4}	0.044	0.049	0.009
Liu and Latham [2009]	17	0.289	0.307	0.897	0.905	0.470

Table S1: Results for the three actual meta-analyses. This table summarizes the findings from the three meta-analyses, focusing on detecting heterogeneity and publication bias. The first column lists the studies by their respective authors. The second column presents the I^2 values. The remaining columns report the *p*-values from five statistical tests: the Q test [Cochran, 1950], posterior-PRP (Q-based), posterior-PRP (Egger-based), Egger's regression test [Egger et al., 1997], and Begg's rank test [Begg and Mazumdar, 1994]. The Q test and posterior-PRP (Q-based) assess heterogeneity. The posterior-PRP (Egger-based), Egger, and Begg tests focus on identifying publication bias.

B Supplementary Figures

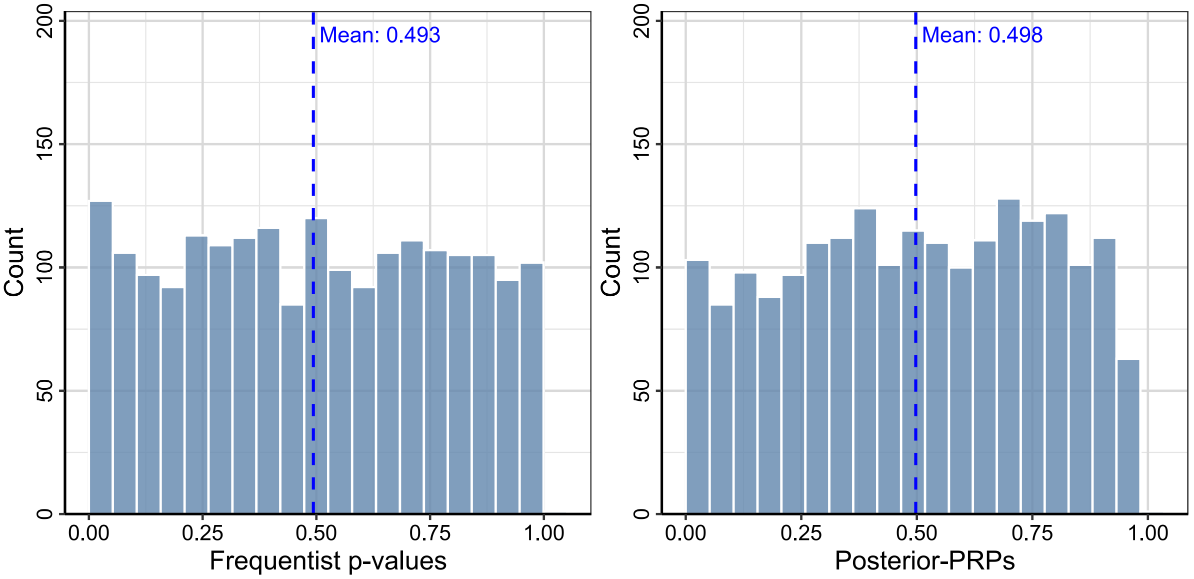

Figure S1: Comparison of frequentist p-values and posterior-PRPs for testing heterogeneity under the reference replicability model with $\eta = 0$. This figure compares the distribution of p-values obtained from Cochran's Q test (panel a) to the distribution of posterior-PRPs (panel b) when the batch effect parameter η is 0.

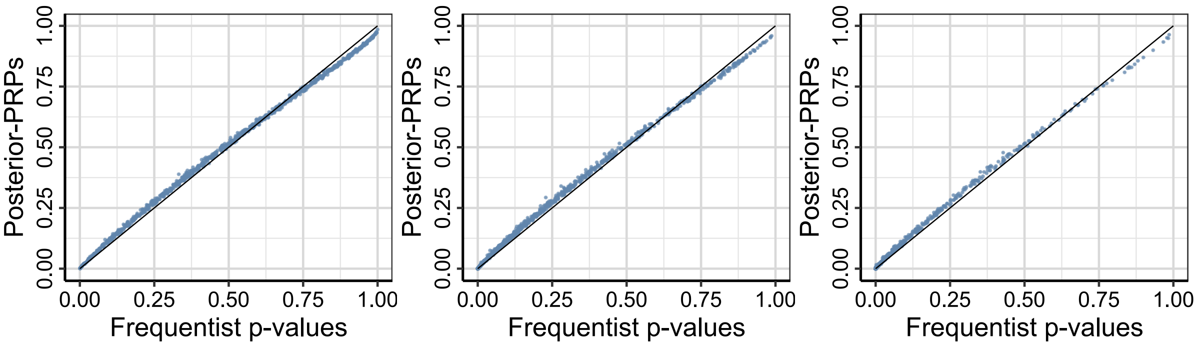

(a) $\eta = 0$ (b) $\eta = 0.4$ (c) $\eta = 0.8$

Figure S2: This figure provides a comparative analysis of frequentist p-values from Cochran's Q test and posterior-PRPs for varying batch effect strengths: (a) $\eta = 0$, (b) $\eta = 0.4$, and (c) $\eta = 0.8$.

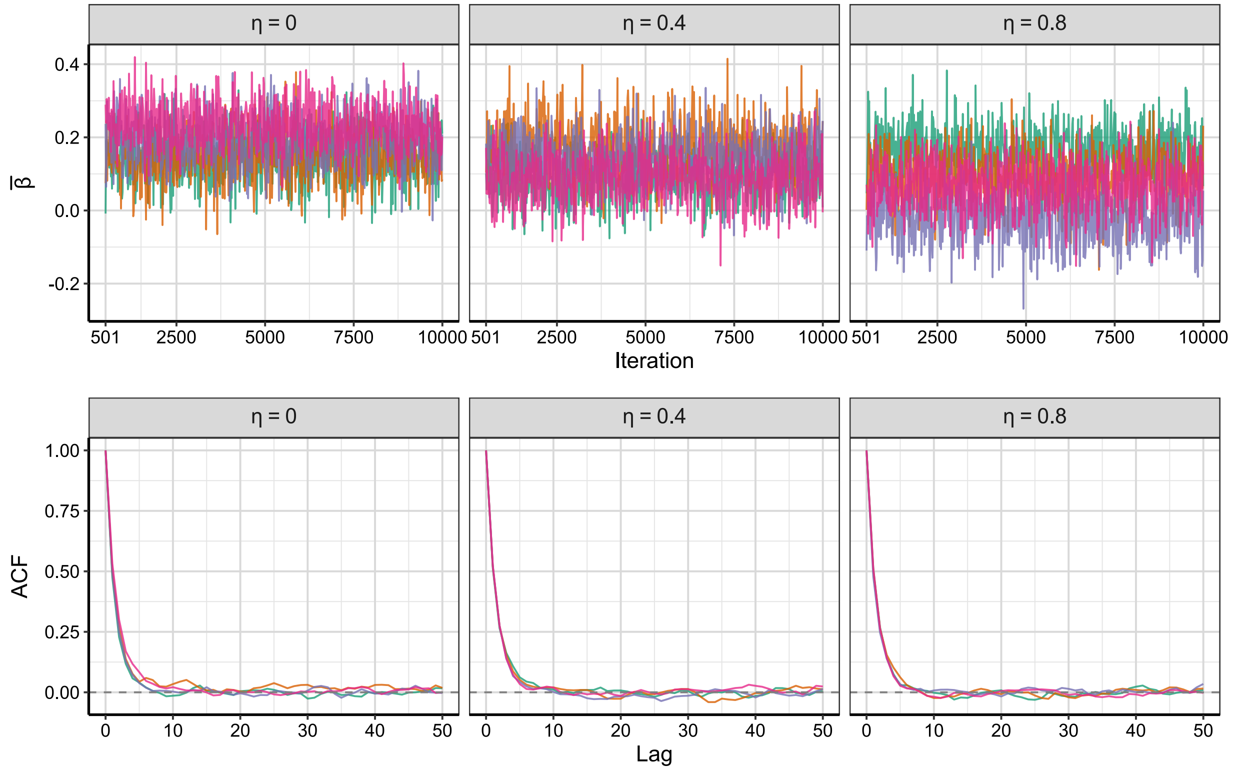

Figure S3: MCMC convergence diagnostics. Top panel is trace plots and bottom panel is ACF curves for four randomly selected simulations (distinguished by color). The results are evaluated across varying batch contamination levels $\eta \in \{0, 0.4, 0.8\}$.

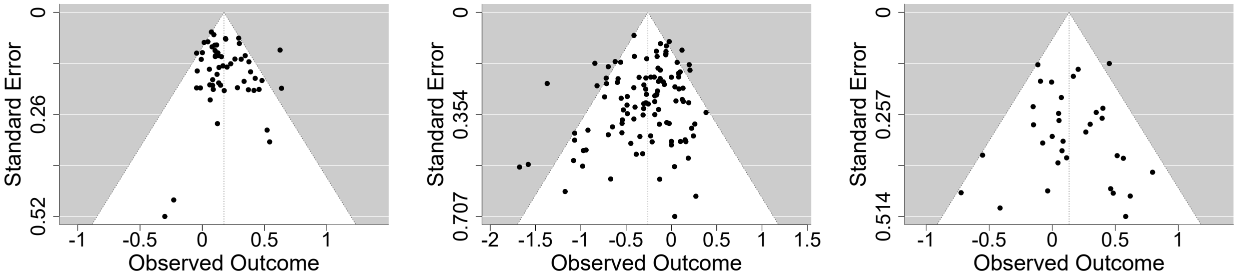

(a) Stead et al. [2012]

(b) Hróbjartsson and Gøtzsche [2010]

(c) Liu and Latham [2009]

Figure S4: Funnel plots of the three actual meta-analyses. This figure presents the funnel plots for three separate meta-analyses assessing publication bias. The unshaded regions represent the 95% confidence intervals, serving as a visual guide for detecting asymmetries that may indicate publication bias. Panel (a) displays the funnel plot from the meta-analysis conducted by Stead et al. [2012] on the effect of nicotine gum on smoking cessation. Panel (b) depicts the funnel plot from Hróbjartsson and Gøtzsche [2010] that examines the effects of placebo interventions. Panel (c) illustrates the funnel plot from the meta-analysis by Liu and Latham [2009], analyzing progressive resistance strength training.

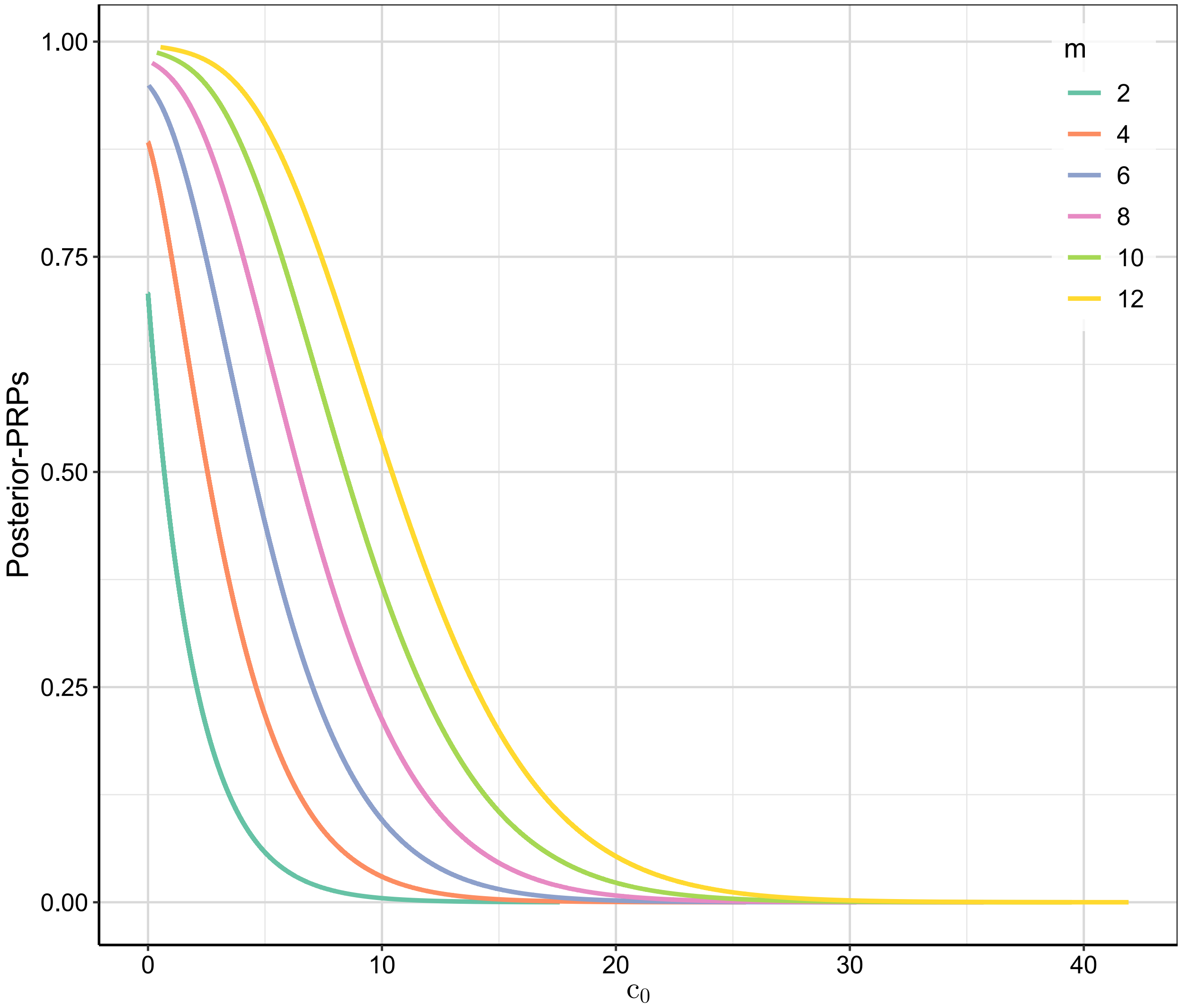

Figure S5: Plot of the function $p_{\text{posterior-PRP}}(c_0)$ (S13) across different values of m under the fixed effect model.

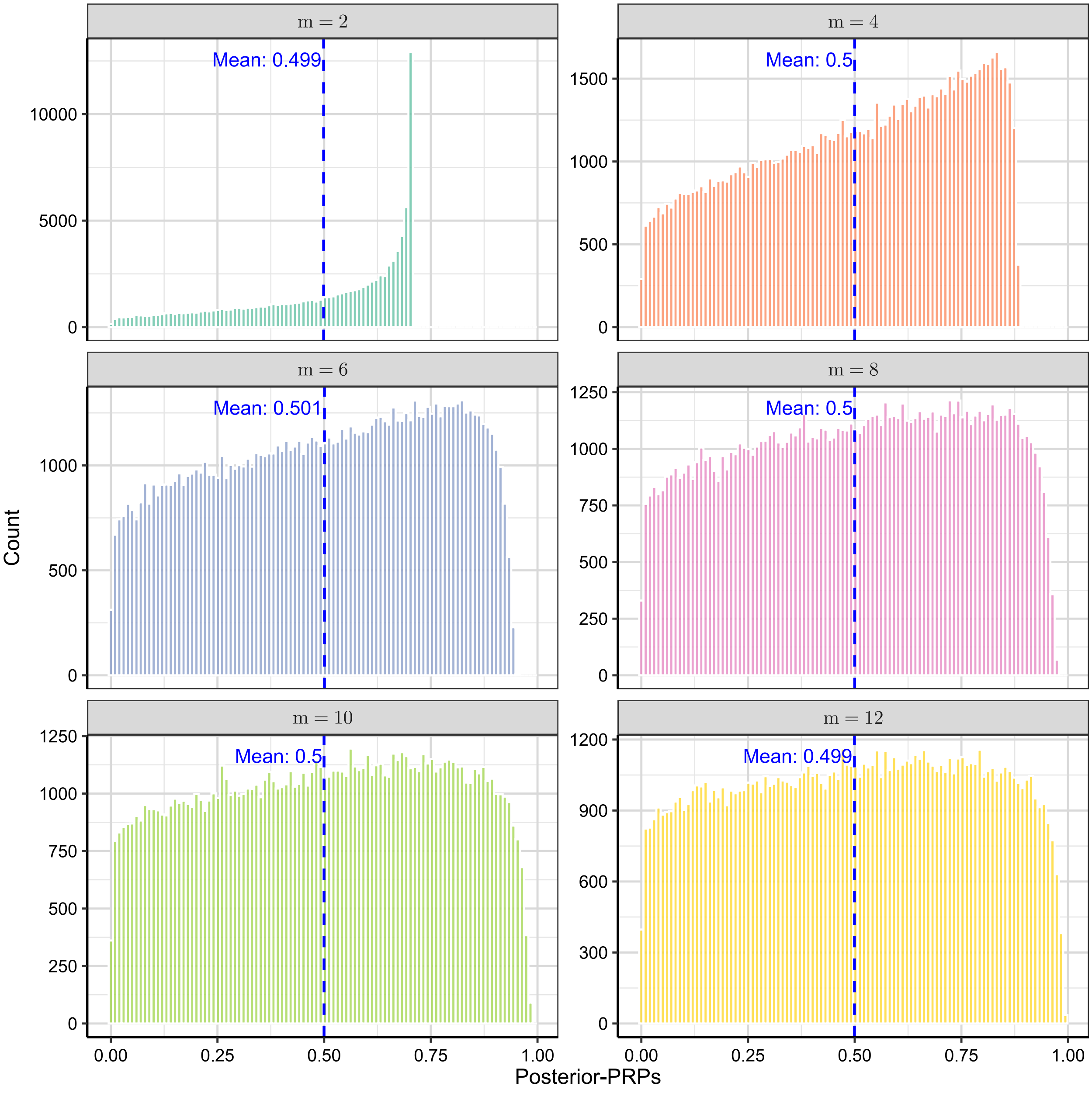

Figure S6: Histogram of $p_{\text{posterior-PRP}}(0)$ for different values of m. The blue dashed lines represent the means in each group.

C Method Details

C.1 Misclassification Probability

Let $\bar{\beta}_1$ and $\bar{\beta}_2$ denote the two distinct grand effects. We assume that the realizations from the two effects follow the normal distributions,

$$\beta_{1,i} \mid \bar{\beta}_1 \sim \mathcal{N}\left(\bar{\beta}_1, \phi^2\right), \quad i = 1, 2, \ldots, \tag{S1}$$
$$\beta_{2,i'} \mid \bar{\beta}_2 \sim \mathcal{N}\left(\bar{\beta}_2, \phi^2\right), \quad i' = 1, 2, \ldots,$$

respectively. Here, $\beta_{1,i}$ and $\beta_{2,i'}$ denote the realizations of grand effects in i-th and i'-th experiment, respectively. Without loss of generality, we assume that the heterogeneity for the two effects is the same and quantified by the variance parameter, ϕ^2.

Based on this generative model, the misclassification probability, $P_{\text{mis}}\left(\bar{\beta}_1, \bar{\beta}_2, \phi^2\right)$, can be computed by

$$P_{\text{mis}}\left(\bar{\beta}_1, \bar{\beta}_2, \phi^2\right)$$
$$= \mathbb{P}(\text{ an effect } \beta \text{ is misclassified from its generative effect })$$
$$= \mathbb{P}\left(\beta \mapsto \bar{\beta}_1 \mid \beta \cong \bar{\beta}_2\right) \mathbb{P}(\beta \cong \bar{\beta}_2) + \mathbb{P}\left(\beta \mapsto \bar{\beta}_2 \mid \beta \cong \bar{\beta}_1\right) \mathbb{P}(\beta \cong \bar{\beta}_1),$$

where $\beta \mapsto \bar{\beta}_1$ represents that β is classified/mapped to $\bar{\beta}_1$, and $\beta \cong \bar{\beta}_1$ denotes that β is generated from $\bar{\beta}_1$. Assuming the exchangeability of the $\bar{\beta}_1$ and $\bar{\beta}_2$, it implies

$$\mathbb{P}(\beta \cong \bar{\beta}_1) = \mathbb{P}(\beta \cong \bar{\beta}_2) = \frac{1}{2}.$$

Furthermore,

$$\begin{aligned}
&\mathbb{P}\left(\beta \mapsto \bar{\beta}_1 \mid \beta \cong \bar{\beta}_2\right) = \mathbb{P}\left(\beta \mapsto \bar{\beta}_2 \mid \beta \cong \bar{\beta}_1\right) \\
&= \int_{-\infty}^{\infty} \mathbb{P}\left(\beta \mapsto \bar{\beta}_1 \mid \beta \cong \bar{\beta}_2, \beta\right) \mathbb{P}\left(\beta \mid \beta \cong \bar{\beta}_2\right) d\beta \\
&= \int_{-\infty}^{\infty} \mathbb{P}\left(\beta \mapsto \bar{\beta}_1 \mid \beta \cong \bar{\beta}_2, \beta\right) \mathcal{N}(\beta; \bar{\beta}_2, \phi^2) d\beta,
\end{aligned}$$

where the notation $\mathcal{N}(x; \mu, \sigma^2)$ denotes the density function of a normal distribution, $\mathcal{N}(\mu, \sigma^2)$, evaluated at x. We have

$$\begin{aligned}
&\mathbb{P}\left(\beta \mapsto \bar{\beta}_1 \mid \beta \cong \bar{\beta}_2, \beta\right) = \frac{\mathbb{P}\left(\beta \mapsto \bar{\beta}_1, \beta \cong \bar{\beta}_2 \mid \beta\right)}{\mathbb{P}\left(\beta \cong \bar{\beta}_2 \mid \beta\right)} \\
=& \frac{\mathbb{P}\left(\beta \cong \bar{\beta}_2 \mid \beta \mapsto \bar{\beta}_1, \beta\right) \mathbb{P}\left(\beta \mapsto \bar{\beta}_1 \mid \beta\right)}{\mathbb{P}\left(\beta \cong \bar{\beta}_2 \mid \beta \mapsto \bar{\beta}_1, \beta\right) \mathbb{P}\left(\beta \mapsto \bar{\beta}_1 \mid \beta\right) + \mathbb{P}\left(\beta \cong \bar{\beta}_2 \mid \beta \mapsto \bar{\beta}_2, \beta\right) \mathbb{P}\left(\beta \mapsto \bar{\beta}_2 \mid \beta\right)} \\
=& \frac{\mathbb{P}\left(\beta \cong \bar{\beta}_2 \mid \beta\right) \mathbb{P}\left(\beta \mapsto \bar{\beta}_1 \mid \beta\right)}{\mathbb{P}\left(\beta \cong \bar{\beta}_2 \mid \beta\right) \mathbb{P}\left(\beta \mapsto \bar{\beta}_1 \mid \beta\right) + \mathbb{P}\left(\beta \cong \bar{\beta}_2 \mid \beta\right) \mathbb{P}\left(\beta \mapsto \bar{\beta}_2 \mid \beta\right)} \\
=& \frac{\frac{1}{2}\mathcal{N}(\beta; \bar{\beta}_1, \phi^2)}{\frac{1}{2}\mathcal{N}\left(\beta; \bar{\beta}_1, \phi^2\right) + \frac{1}{2}\mathcal{N}(\beta; \bar{\beta}_2, \phi^2)} \\
=& \frac{\mathcal{N}(\beta; \bar{\beta}_1, \phi^2)}{\mathcal{N}(\beta; \bar{\beta}_1, \phi^2) + \mathcal{N}(\beta; \bar{\beta}_2, \phi^2)}.
\end{aligned}$$

Then, we have

$$\begin{aligned}
&\mathbb{P}\left(\beta \mapsto \bar{\beta}_1 \mid \beta \cong \bar{\beta}_2\right) = \mathbb{P}\left(\beta \mapsto \bar{\beta}_2 \mid \beta \cong \bar{\beta}_1\right) \\
&= \int_{-\infty}^{\infty} \frac{\mathcal{N}(\beta; \bar{\beta}_1, \phi^2)\mathcal{N}(\beta; \bar{\beta}_2, \phi^2)}{\mathcal{N}(\beta; \bar{\beta}_1, \phi^2) + \mathcal{N}(\beta; \bar{\beta}_2, \phi^2)} d\beta.
\end{aligned}$$

Altogether, it can be shown that

$$P_{\text{mis}}\left(\bar{\beta}_1, \bar{\beta}_2, \phi^2\right) = \int_{-\infty}^{\infty} \frac{\mathcal{N}(\beta; \bar{\beta}_1, \phi^2)\mathcal{N}(\beta; \bar{\beta}_2, \phi^2)}{\mathcal{N}(\beta; \bar{\beta}_1, \phi^2) + \mathcal{N}(\beta; \bar{\beta}_2, \phi^2)} d\beta. \tag{S2}$$

Straightforward algebra shows that the integral in equation (S2) can be equivalently ex-

pressed as an expectation to a standard normal random variable, Z, i.e.,

$$P_{\text{mis}}\left(\bar{\beta}_1, \bar{\beta}_2, \phi^2\right) = \mathbb{E}_Z\left[\left(1 + \exp\left[\frac{1}{2}\left(\frac{\left|\bar{\beta}_1 - \bar{\beta}_2\right|}{\phi}\right)^2 + \frac{\left|\bar{\beta}_1 - \bar{\beta}_2\right|}{\phi} Z\right]\right)^{-1}\right]. \tag{S3}$$

Equation (S3) implies that the misclassification probability is a monotonic function that depends only on the quantity $\frac{\left|\bar{\beta}_1 - \bar{\beta}_2\right|}{\phi}$. This property is formally proved in Section C.2.

Under this exchangeable normal model implementation of the distinguishability criterion, the fixed effect model is represented when $\phi \to 0$ and $\bar{\beta}_1 \neq \bar{\beta}_2$, which yields $\frac{\left|\bar{\beta}_1 - \bar{\beta}_2\right|}{\phi} \to \infty$ and $P_{\text{mis}} \to 0$, whereas when $\frac{\left|\bar{\beta}_1 - \bar{\beta}_2\right|}{\phi} \to 0, P_{\text{mis}} \to \frac{1}{2}$. In the fixed effect model, $P_{\text{mis}} \to 0$ as there is no heterogeneity, eliminating misclassification. Conversely, when $\frac{\left|\bar{\beta}_1 - \bar{\beta}_2\right|}{\phi} \to 0$, the two grand effects are nearly indistinguishable, leading to random misclassification between them with $P_{\text{mis}} \to \frac{1}{2}$.

The most important utility of equation (S3) is to allow defining an acceptable level of heterogeneity, ϕ, by an easy-to-interpret probability, P_{mis}. That is, given $\bar{\beta}_1, \bar{\beta}_2$ and P_{mis}, parameter ϕ in the exchangeable normal model is determined.

In the special case that $\bar{\beta}_2 = 0$ is set as a natural reference point, we remove the index of $\bar{\beta}_1$ and denote it by $\bar{\beta}$. Let $k := \phi/|\bar{\beta}|$. Under this special case and the re-parameterization, the misclassification probability becomes a univariate function of k, i.e.,

$$P_{\text{mis}}(k) = \mathbb{E}_Z\left[\left(1 + \exp\left[\frac{1}{2}\left(\frac{1}{k}\right)^2 + \frac{1}{k} Z\right]\right)^{-1}\right]. \tag{S4}$$

Moreover, based on model (S1) and $\phi^2 = k^2\bar{\beta}^2$, the sampling from $\bar{\beta}$ is

$$\beta_i \sim \mathcal{N}(\bar{\beta}, k^2\bar{\beta}^2), \quad i = 1, 2, \ldots,$$

where the variance is the function of the mean.

C.2 Monotonicity of Misclassification Probability

Define

$$g_\delta(z) := \sigma(\frac{1}{2}\delta^2 + \delta z) = (1 + \exp\{\frac{1}{2}\delta^2 + \delta z\})^{-1} \in (0,1),$$

where $\delta = |\bar{\beta}_1 - \bar{\beta}_2|/\phi$ and $\sigma(x) = 1/(1+e^x)$ is logistic function. So, we have

$$P_{\text{mis}}(\delta) = \mathbb{E}_Z[g_\delta(Z)], \quad \delta \geq 0.$$

For logistic function, we have

$$\frac{\partial \sigma(x)}{\partial x} = -\sigma(x)(1-\sigma(x)) \quad \text{and} \quad \sigma(x)(1-\sigma(x)) \leq \frac{1}{4}.$$

Then

$$\frac{\partial g_\delta(z)}{\partial \delta} = -\,(\delta + z)\, g_\delta(z)\big(1 - g_\delta(z)\big).$$

Since $|\partial g_\delta(z)/\partial \delta| \leq \frac{1}{4}(|\delta| + |z|)$, it is integrable. For $h_\delta(z) = g_\delta(z)(1 - g_\delta(z))$, we have

$$\frac{\partial g_\delta(z)}{\partial z} = -\delta\, h_\delta(z), \qquad \frac{\partial h_\delta(z)}{\partial z} = -\delta\, h_\delta(z)\,(1 - 2g_\delta(z)).$$

Stein's lemma ($\mathbb{E}[Z\, h_\delta(Z)] = \mathbb{E}[\frac{\partial h_\delta(Z)}{\partial Z}]$, h_δ is a differentiable function) yields

$$\mathbb{E}[Z\, h_\delta(Z)] = -\delta\, \mathbb{E}[h_\delta(Z)\,(1 - 2g_\delta(Z))].$$

We now compute

$$
\begin{aligned}
\frac{\partial P_{\text{mis}}(\delta)}{\partial \delta} &= \mathbb{E}_Z\left[\frac{\partial g_\delta(Z)}{\partial \delta}\right] \\
&= -\mathbb{E}_Z\big[(\delta + Z) g_\delta(Z)\big(1 - g_\delta(Z)\big)\big] \\
&= -\mathbb{E}_Z\big[(\delta + Z) h_\delta(Z)\big] \\
&= -\delta\, \mathbb{E}_Z[h_\delta(Z)] - \mathbb{E}_Z[Z\, h_\delta(Z)] \\
&= -\delta\, \mathbb{E}_Z[h_\delta(Z)] + \delta\, \mathbb{E}_Z[h_\delta(Z)\,(1 - 2g_\delta(Z))] \\
&= -2\delta\, \mathbb{E}_Z[g_\delta(Z)^2(1 - g_\delta(Z))].
\end{aligned}
$$

Because $g_\delta(Z) \in (0,1)$ and $\delta \geq 0$, the expectation is negative. Hence

$$
\frac{\partial P_{\text{mis}}(\delta)}{\partial \delta} \leq 0 \quad \forall\, \delta \geq 0.
$$

P_{mis} is monotone decreasing in $|\bar{\beta}_1 - \bar{\beta}_2|/\phi$.

C.3 Derivation of Directional Consistency Probability

We provide a rigorous derivation showing that our distinguishability criterion inherently enforces directional consistency with high probability. Under the reference replicability model, the effect size β_j follows $\beta_j \mid \bar{\beta}, k \sim \mathcal{N}(\bar{\beta}, k^2\bar{\beta}^2)$. The probability of a sign flip (directional inconsistency) corresponds to the event where β_j and $\bar{\beta}$ have opposite signs. We have

$$
\begin{aligned}
\mathbb{P}(\text{sign}(\beta_j) \neq \text{sign}(\bar{\beta}) \mid k) &= \mathbb{P}(\beta_j < 0 \mid \bar{\beta} > 0)\mathbb{P}(\bar{\beta} > 0) + \mathbb{P}(\beta_j > 0 \mid \bar{\beta} < 0)\mathbb{P}(\bar{\beta} < 0) \\
&= \mathbb{P}\left(\frac{\beta_j - \bar{\beta}}{k\bar{\beta}} < \frac{0 - \bar{\beta}}{k\bar{\beta}}\right)\mathbb{P}(\bar{\beta} > 0) + \mathbb{P}\left(\frac{\beta_j - \bar{\beta}}{k\bar{\beta}} < \frac{0 - \bar{\beta}}{k\bar{\beta}}\right)\mathbb{P}(\bar{\beta} < 0) \\
&= \mathbb{P}\left(Z < -\frac{1}{k}\right)\big[\mathbb{P}(\bar{\beta} > 0) + \mathbb{P}(\bar{\beta} < 0)\big] = \Phi\left(-\frac{1}{k}\right),
\end{aligned}
$$

where $Z \sim \mathcal{N}(0,1)$ and $\Phi(\cdot)$ is the cumulative distribution function (CDF) of $\mathcal{N}(0,1)$.

The sign-reversal probability depends only on k. Since k is monotone increasing in P_{mis}, our default choice of $P_{\text{mis}} \leq 0.05$ implies $k \leq f^{-1}(0.05) \approx 0.2727$, where f^{-1} denote the inverse mapping of function (S4). The sign-reversal probability is bounded by $\Phi(-1/0.2727) \approx 0.00012$. This means that under our distinguishability criterion, the probability of a sign flip is approximately 0.012%, ensuring that directional consistency is maintained with high probability.

C.4 Sampling Method

In this section, we will detail how each quantity in algorithm 1 is sampled. Recall that our reference replicability model is

$$
\begin{aligned}
\bar{\beta} &\sim 1 \\
\beta_j \mid \bar{\beta}, k &\sim \mathcal{N}(\bar{\beta}, k^2\bar{\beta}^2) \\
\hat{\beta}_j \mid \beta_j &\sim \mathcal{N}(\beta_j, \hat{\sigma}_j^2), \quad j = 1, \dots, m,
\end{aligned}
$$

where $\bar{\beta}$ is the grand effect, β_j is realization of grand effect in each experiment, $\hat{\beta}_j$ is the observed effect, and $\hat{\sigma}_j^2$ is the observed variance of effect. The number of experiments/studies is m.

Based on the posterior predictive checking framework, all unknown quantities are sampled conditioning on observed data $\hat{\beta}_j, j = 1, \dots, m$. The parameters $\bar{\beta}$ and k are sampled using the Metropolis-Hastings (MH) algorithm, a type of Markov Chain Monte Carlo (MCMC) method [Hastings, 1970, Metropolis et al., 1953]. Subsequently, conditioning on $\bar{\beta}$, k, and observed data $\hat{\beta}_j$, we can explicitly calculate that $\beta_j, j = 1, \dots, m$ follows a normal distribution. The detailed algorithm is summarized in algorithm S1

C.4.1 Sample Misclassification Probability

Users can also specify the distribution of P_{mis}, such as Uniform$[0, 0.05]$, a Beta distribution truncated to the $[0, 0.05]$ interval, or a constant value. We propose sampling $-\log_{10}(P_{\text{mis}})$ from a Uniform$[-\log_{10}(0.05), 10]$ distribution. Typically, the more the sampling distribution of P_{mis} is concentrated around 0, the lower the tolerated heterogeneity level. Consequently, the resulting posterior-PRP is less likely to support the replicability. After sampling P_{mis}, we can compute k according to equation (S4), that is, $k = f^{-1}\left(P_{\text{mis}}\right)$, where f^{-1} denote the inverse mapping of function (S4).

C.4.2 Sample $\bar{\beta}$

Given $\bar{\beta}$ and k, the density of $\hat{\boldsymbol{\beta}} = (\hat{\beta}_1, \ldots, \hat{\beta}_m)$ is

$$p\left(\hat{\boldsymbol{\beta}} \mid \bar{\beta}, k\right) \propto \prod_{j=1}^{m} \frac{1}{\sqrt{k^2\bar{\beta}^2 + \hat{\sigma}_j^2}} \exp\left[-\frac{1}{2\left(k^2\bar{\beta}^2 + \hat{\sigma}_j^2\right)}\left(\hat{\beta}_j - \bar{\beta}\right)^2\right]. \tag{S5}$$

Given $\hat{\boldsymbol{\beta}}, k$, the density of $\bar{\beta}$ is

$$\begin{aligned} p\left(\bar{\beta} \mid \hat{\boldsymbol{\beta}}, k\right) &\propto p(\bar{\beta}) \cdot p(\hat{\boldsymbol{\beta}} \mid \bar{\beta}, k) \\ &\propto \prod_{j=1}^{m} \frac{1}{\sqrt{k^2\bar{\beta}^2 + \hat{\sigma}_j^2}} \exp\left[-\frac{1}{2\left(k^2\bar{\beta}^2 + \hat{\sigma}_j^2\right)}\left(\hat{\beta}_j - \bar{\beta}\right)^2\right] \\ &\triangleq g\left(\bar{\beta} \mid \hat{\boldsymbol{\beta}}, k\right). \end{aligned} \tag{S6}$$

We can sample $\bar{\beta}$ by MH given $\hat{\boldsymbol{\beta}}, k$. The proposed distribution is

$$q(\bar{\beta}^* \mid \hat{\boldsymbol{\beta}}) = \mathcal{N}\left(\text{mean}(\hat{\boldsymbol{\beta}}), k_{\max}^2[\text{mean}(\hat{\boldsymbol{\beta}})]^2 + \text{mean}(\hat{\boldsymbol{\Sigma}})\right),$$

where $\hat{\boldsymbol{\Sigma}} = (\hat{\sigma}_1^2, \ldots, \hat{\sigma}_m^2)$, $\text{mean}(\hat{\boldsymbol{\beta}}) = \frac{1}{m}\sum_{j=1}^{m}\hat{\beta}_j$, $\text{mean}(\hat{\boldsymbol{\Sigma}}) = \frac{1}{m}\sum_{j=1}^{m}\hat{\sigma}_j^2$, $k_{\max} = f^{-1}(0.05)$,

and we use the upper mark $*$ to represent the candidate value. The lower marker $(i-1)$ is used to represent the value at the $(i-1)$-th iteration in the MH algorithm.

The MH acceptance ratio is

$$a = \min\left(1, \frac{g(\bar{\beta}^* \mid \hat{\boldsymbol{\beta}}, k^*) \cdot q(\bar{\beta}_{(i-1)} \mid \hat{\boldsymbol{\beta}})}{g(\bar{\beta}_{(i-1)} \mid \hat{\boldsymbol{\beta}}, k_{(i-1)}) \cdot q(\bar{\beta}^* \mid \hat{\boldsymbol{\beta}})}\right).$$

Remark S1 (Posterior propriety under a flat prior)**.** *Fix $k \geq 0$ and assume $\hat{\sigma}_j^2 > 0$ for all $j = 1, \ldots, m$. Under the flat prior $p(\bar{\beta}) \propto 1$, the posterior density $p(\bar{\beta} \mid \hat{\boldsymbol{\beta}}, k) \propto p(\hat{\boldsymbol{\beta}} \mid \bar{\beta}, k)$ is proper (i.e., normalizable) for any $m \geq 2$.*

Proof. *Let*

$$\phi(\bar{\beta}) := \prod_{j=1}^{m} \frac{1}{\sqrt{k^2\bar{\beta}^2 + \hat{\sigma}_j^2}} \exp\left[-\frac{(\hat{\beta}_j - \bar{\beta})^2}{2\left(k^2\bar{\beta}^2 + \hat{\sigma}_j^2\right)}\right],$$

which is the unnormalized posterior kernel under $p(\bar{\beta}) \propto 1$ and $\phi(\bar{\beta}) \propto p(\hat{\boldsymbol{\beta}} \mid \bar{\beta}, k)$. Since the exponential terms are bounded by 1, we have, for all $\bar{\beta} \in \mathbb{R}$,

$$\phi(\bar{\beta}) \leq \prod_{j=1}^{m} \frac{1}{\sqrt{k^2\bar{\beta}^2 + \hat{\sigma}_j^2}}. \tag{S7}$$

We bound $\int_{\mathbb{R}} \phi(\bar{\beta})\, d\bar{\beta}$ by considering $|\bar{\beta}| \leq 1$ and $|\bar{\beta}| > 1$. First, on the set $|\bar{\beta}| \leq 1$, we use $k^2\bar{\beta}^2 + \hat{\sigma}_j^2 \geq \hat{\sigma}_j^2$ to obtain

$$\phi(\bar{\beta}) \leq \prod_{j=1}^{m} \hat{\sigma}_j^{-1},$$

and hence

$$\int_{|\bar{\beta}| \leq 1} \phi(\bar{\beta})\, d\bar{\beta} \leq 2 \prod_{j=1}^{m} \hat{\sigma}_j^{-1} < \infty.$$

Next, consider on the set $|\bar{\beta}| > 1$. If $k = 0$, then $\phi(\bar{\beta})$ reduces to a product proportional to normal densities with fixed variances $\hat{\sigma}_j^2$, and thus is integrable. If $k > 0$, then the inequality

$k^2\bar{\beta}^2 + \hat{\sigma}_j^2 \geq k^2\bar{\beta}^2$ implies

$$\phi(\bar{\beta}) \leq \prod_{j=1}^{m} \frac{1}{k|\bar{\beta}|} = k^{-m}|\bar{\beta}|^{-m},$$

and therefore

$$\int_{|\bar{\beta}|>1} \phi(\bar{\beta})\, d\bar{\beta} \leq 2k^{-m} \int_1^{\infty} x^{-m}\, dx = \frac{2k^{-m}}{m-1} < \infty \quad \text{for } m > 1.$$

74 *Combining the two parts gives $\int_{\mathbb{R}} \phi(\bar{\beta})\, d\bar{\beta} < \infty$ whenever $m \geq 2$. Given that study number*
75 *m is naturally greater than 1 within our replicability framework, this requirement is trivially*
76 *met. Thus the posterior $p(\bar{\beta} \mid \hat{\boldsymbol{\beta}}, k)$ is proper under the flat prior.* □

77 C.4.3 Sample β_j

Given $\hat{\beta}_j, \bar{\beta}$, and $k, j = 1, \ldots, m$, the density of β_j is

$$\begin{aligned} p\left(\beta_j \mid \hat{\beta}_j, \bar{\beta}, k\right) &\propto p(\hat{\beta}_j \mid \beta_j) \cdot p\left(\beta_j \mid \bar{\beta}, k\right) \\ &\propto \exp\left[-\frac{1}{2\hat{\sigma}_j^2}\left(\hat{\beta}_j - \beta_j\right)^2\right] \cdot \exp\left[-\frac{1}{2k^2\bar{\beta}^2}\left(\beta_j - \bar{\beta}\right)^2\right] \\ &\propto \exp\left[-\frac{1}{2}\beta_j^2\left(\frac{1}{k^2\bar{\beta}^2} + \frac{1}{\hat{\sigma}_j^2}\right) + \beta_j\left(\frac{\bar{\beta}}{k^2\bar{\beta}^2} + \frac{\hat{\beta}_j}{\hat{\sigma}_j^2}\right)\right]. \end{aligned}$$

78 Thus,

$$\beta_j \mid \hat{\beta}_j, \bar{\beta}, k \sim \mathcal{N}\left(\left(\frac{1}{k^2\bar{\beta}^2} + \frac{1}{\hat{\sigma}_j^2}\right)^{-1}\left(\frac{\bar{\beta}}{k^2\bar{\beta}^2} + \frac{\hat{\beta}_j}{\hat{\sigma}_j^2}\right),\ \left(\frac{1}{k^2\bar{\beta}^2} + \frac{1}{\hat{\sigma}_j^2}\right)^{-1}\right). \tag{S8}$$

To avoid numerical instability when k or $\bar{\beta}$ approaches zero, the posterior distribution is algebraically simplified by eliminating the inverse terms in the denominator. We have

$$\left(\frac{1}{k^2\bar{\beta}^2} + \frac{1}{\hat{\sigma}_j^2}\right)^{-1} = \left(\frac{\hat{\sigma}_j^2 + k^2\bar{\beta}^2}{k^2\bar{\beta}^2\hat{\sigma}_j^2}\right)^{-1} = \frac{k^2\bar{\beta}^2\hat{\sigma}_j^2}{\hat{\sigma}_j^2 + k^2\bar{\beta}^2}$$

and

$$\left(\frac{1}{k^2\bar{\beta}^2}+\frac{1}{\hat{\sigma}_j^2}\right)^{-1}\left(\frac{\bar{\beta}}{k^2\bar{\beta}^2}+\frac{\hat{\beta}_j}{\hat{\sigma}_j^2}\right)=\frac{k^2\bar{\beta}^2\hat{\sigma}_j^2}{\hat{\sigma}_j^2+k^2\bar{\beta}^2}\cdot\left(\frac{\bar{\beta}}{k^2\bar{\beta}^2}+\frac{\hat{\beta}_j}{\hat{\sigma}_j^2}\right)=\frac{\hat{\sigma}_j^2\bar{\beta}+k^2\bar{\beta}^2\hat{\beta}_j}{\hat{\sigma}_j^2+k^2\bar{\beta}^2}.$$

We get

$$\beta_j \mid \hat{\beta}_j, \bar{\beta}, k \sim \mathcal{N}\left(\frac{\hat{\sigma}_j^2\bar{\beta}+k^2\bar{\beta}^2\hat{\beta}_j}{\hat{\sigma}_j^2+k^2\bar{\beta}^2}, \frac{k^2\bar{\beta}^2\hat{\sigma}_j^2}{\hat{\sigma}_j^2+k^2\bar{\beta}^2}\right). \tag{S9}$$

C.4.4 Sample $\hat{\beta}_j'$ and Construct Test Statistics

We sample $\hat{\beta}_j' \mid \beta_j \sim \mathcal{N}\left(\beta_j, \hat{\sigma}_j^2\right), j = 1, \ldots, m$.

Motivated by Cochran's Q statistic [Cochran, 1950, Patil, 1975], the test quantity $T(\boldsymbol{X}, \boldsymbol{\theta}) = T(\hat{\boldsymbol{\beta}}, (\bar{\beta}, k))$ we use by default in our proposed reference replicability model is

$$T(\hat{\boldsymbol{\beta}}, (\bar{\beta}, k)) = \sum_{j=1}^{m} \tilde{w}_j \left(\hat{\beta}_j - \bar{\beta}\right)^2, \text{ where } \tilde{w}_j = \frac{1}{\hat{\sigma}_j^2 + k^2\bar{\beta}^2}.$$

D Posterior-PRPs in Fixed Effect Model

D.1 Model

For the posterior-PRPs in fixed effect model ($k = 0$), we have the hierarchical model with m studies

$$\bar{\beta} \sim 1 \quad \text{and} \quad \hat{\beta}_j \mid \bar{\beta} \sim \mathcal{N}(\bar{\beta}, \hat{\sigma}_j^2), \quad \text{for } j = 1, \ldots, m.$$

Given the observed effect sizes $\hat{\boldsymbol{\beta}} = (\hat{\beta}_1, \ldots, \hat{\beta}_m)$ and variances $\hat{\boldsymbol{\Sigma}} = (\hat{\sigma}_1^2, \ldots, \hat{\sigma}_m^2)$, the

Algorithm S1 Posterior distributions via Metropolis-Hastings MCMC

procedure Metropolis-Hastings MCMC

  Input: N (total MCMC iterations), r (burn-in rate), $\hat{\boldsymbol{\beta}} = \left(\hat{\beta}_1, \ldots, \hat{\beta}_m\right)$,

  $\hat{\boldsymbol{\Sigma}} = (\hat{\sigma}_1^2, \ldots, \hat{\sigma}_m^2)$, and user-specified distribution for P_{mis}, by default, $-\log_{10}(P_{\text{mis}})$ is from a Uniform$[-\log_{10}(0.05), 10]$

  Initiate: $\bar{\beta}_{(0)} \leftarrow \hat{\beta}_1, k_{(0)} \leftarrow k_{\max}, \text{count} \leftarrow 0$

  for $i = 1$ to N **do**

    Sample $\bar{\beta}^* \sim \mathcal{N}(\text{mean}(\hat{\boldsymbol{\beta}}), k_{\max}^2[\text{mean}(\hat{\boldsymbol{\beta}})]^2 + \text{mean}(\hat{\boldsymbol{\Sigma}}))$

    Sample P_{mis}^* and compute $k^* = f^{-1}(P_{\text{mis}}^*)$

    Propose $(\bar{\beta}^*, k^*)$ and evaluate the M-H acceptance ratio a

$$a = \min\left(1, \frac{g(\bar{\beta}^* \mid \hat{\boldsymbol{\beta}}, k^*) \cdot q(\bar{\beta}_{(i-1)} \mid \hat{\boldsymbol{\beta}})}{g(\bar{\beta}_{(i-1)} \mid \hat{\boldsymbol{\beta}}, k_{(i-1)}) \cdot q(\bar{\beta}^* \mid \hat{\boldsymbol{\beta}})}\right)$$

    Generate a uniform random number $u \sim \text{Uniform}[0, 1]$

    if $a \geq u$ **then**

      $\bar{\beta}_{(i)} \leftarrow \bar{\beta}^*, k_{(i)} \leftarrow k^*$

    else

      $\bar{\beta}_{(i)} \leftarrow \bar{\beta}_{(i-1)}, k_{(i)} \leftarrow k_{(i-1)}$

    end if

    if $i > N \times r$ **then**

      Sample $\beta_j \sim \mathcal{N}\left(\frac{\hat{\sigma}_j^2 \bar{\beta}_{(i)} + k_{(i)}^2 \bar{\beta}_{(i)}^2 \hat{\beta}_j}{\hat{\sigma}_j^2 + k_{(i)}^2 \bar{\beta}_{(i)}^2}, \frac{k_{(i)}^2 \bar{\beta}_{(i)}^2 \hat{\sigma}_j^2}{\hat{\sigma}_j^2 + k_{(i)}^2 \bar{\beta}_{(i)}^2}\right)$

      Sample $\hat{\beta}_j' \sim \mathcal{N}\left(\beta_j, \hat{\sigma}_j^2\right), \quad j = 1, \ldots, m$

      Compute test statistic $T(\hat{\boldsymbol{\beta}}', (\bar{\beta}_{(i)}, k_{(i)}))$

      if $T(\hat{\boldsymbol{\beta}}', (\bar{\beta}_{(i)}, k_{(i)})) \geq T(\hat{\boldsymbol{\beta}}, (\bar{\beta}_{(i)}, k_{(i)}))$ **then**

        $\text{count} \leftarrow \text{count} + 1$

      end if

    end if

  end for

  Compute one-sided p-value: $p_{\text{posterior-PRP}} = \frac{\text{count}}{N - N \times r}$

  return $p_{\text{posterior-PRP}}$

end procedure

posterior distribution of $\bar{\beta}$ is

$$
\begin{aligned}
p\left(\bar{\beta} \mid \hat{\boldsymbol{\beta}}\right) &\propto p(\bar{\beta}) \cdot p(\hat{\boldsymbol{\beta}} \mid \bar{\beta}) \\
&\propto \prod_{j=1}^{m} \exp\left[-\frac{1}{2\hat{\sigma}_j^2}\left(\hat{\beta}_j - \bar{\beta}\right)^2\right] \\
&\propto \exp\left[-\frac{1}{2}\bar{\beta}^2\left(\sum_{j=1}^{m}\frac{1}{\hat{\sigma}_j^2}\right) + \bar{\beta}\left(\sum_{j=1}^{m}\frac{\hat{\beta}_j}{\hat{\sigma}_j^2}\right)\right].
\end{aligned}
$$

Comparing this with the density function of a normal distribution, we have

$$
\bar{\beta} \mid \hat{\boldsymbol{\beta}} \sim \mathcal{N}\left(\mu_{\bar{\beta}}, V_{\bar{\beta}}\right), \tag{S10}
$$

where

$$
\mu_{\bar{\beta}} = \frac{\sum_{j=1}^{m} w_j \hat{\beta}_j}{\sum_{j=1}^{m} w_j}, \quad V_{\bar{\beta}} = \left(\sum_{j=1}^{m} w_j\right)^{-1}, \text{ and } w_j = \frac{1}{\hat{\sigma}_j^2}.
$$

The replicated data $\hat{\beta}_j'$ is from the posterior predictive distribution

$$
\hat{\beta}_j' \mid \bar{\beta} \sim \mathcal{N}(\bar{\beta}, \hat{\sigma}_j^2), \quad j = 1, \ldots, m.
$$

The test statistic is defined as $T(\hat{\boldsymbol{\beta}}, \bar{\beta}) = \sum_{j=1}^{m} w_j(\hat{\beta}_j - \bar{\beta})^2$.

Recall that R_0 means that the probability is evaluated under the reference replicability model, then a posterior-PRP is defined as

$$
p_{\text{posterior-PRP}} = \mathbb{P}(T(\hat{\boldsymbol{\beta}}', \bar{\beta}) \geq T(\hat{\boldsymbol{\beta}}, \bar{\beta}) \mid \hat{\boldsymbol{\beta}}, R_0).
$$

D.2 Derivation for Posterior-PRPs

In this section, we provide a derivation of the posterior-PRP for the general case.

D.2.1 Distribution of $T(\hat{\boldsymbol{\beta}}, \bar{\beta})$ given $\hat{\boldsymbol{\beta}}$

Given that $\bar{\beta} \mid \hat{\boldsymbol{\beta}} \sim \mathcal{N}(\mu_{\bar{\beta}}, V_{\bar{\beta}})$, and the observed $\hat{\boldsymbol{\beta}}$ is fixed, the randomness in $T = T(\hat{\boldsymbol{\beta}}, \bar{\beta})$ arises from $\bar{\beta}$. We have

$$
\begin{aligned}
T &= \sum_{j=1}^{m} w_j (\hat{\beta}_j - \bar{\beta})^2 \\
&= \sum_{j=1}^{m} w_j \left((\hat{\beta}_j - \mu_{\bar{\beta}}) - (\bar{\beta} - \mu_{\bar{\beta}}) \right)^2 \\
&= \sum_{j=1}^{m} w_j (\hat{\beta}_j - \mu_{\bar{\beta}})^2 - 2(\bar{\beta} - \mu_{\bar{\beta}}) \sum_{j=1}^{m} w_j (\hat{\beta}_j - \mu_{\bar{\beta}}) + (\bar{\beta} - \mu_{\bar{\beta}})^2 \sum_{j=1}^{m} w_j.
\end{aligned}
$$

Since $\mu_{\bar{\beta}} = \frac{\sum_{j=1}^{m} w_j \hat{\beta}_j}{\sum_{j=1}^{m} w_j}$, it follows that

$$
\sum_{j=1}^{m} w_j (\hat{\beta}_j - \mu_{\bar{\beta}}) = 0.
$$

Thus, the middle term vanishes. We now have

$$
T = c_0 + (\bar{\beta} - \mu_{\bar{\beta}})^2 V_{\bar{\beta}}^{-1},
$$

where $c_0 = \sum_{j=1}^{m} w_j (\hat{\beta}_j - \mu_{\bar{\beta}})^2$ is a constant given $\hat{\boldsymbol{\beta}}$. Since $\bar{\beta} \sim \mathcal{N}(\mu_{\bar{\beta}}, V_{\bar{\beta}})$, we have $T = c_0 + \mathcal{N}(0,1)^2$, so

$$
T - c_0 \sim \chi_1^2.
$$

D.2.2 Distribution of $T(\hat{\boldsymbol{\beta}}', \bar{\beta})$

Denote $\hat{\boldsymbol{\beta}} = (\hat{\boldsymbol{\beta}}_1, \ldots, \hat{\boldsymbol{\beta}}_m)$. Since the replicated data $\hat{\beta}_j' \sim \mathcal{N}(\bar{\beta}, \hat{\sigma}_j^2), j = 1, \ldots, m$, we have

$$
T' = T(\hat{\boldsymbol{\beta}}', \bar{\beta}) = \sum_{j=1}^{m} \left(\frac{\hat{\beta}_j' - \bar{\beta}}{\hat{\sigma}_j} \right)^2 \sim \chi_m^2.
$$

D.2.3 Computing $p_{\textbf{posterior-PRP}}$

In computing the posterior-PRP, we calculate the probability that T' is greater than T given observed $\hat{\boldsymbol{\beta}}$ and under the reference replicability model. Specifically, we have

$$
\begin{aligned}
p_{\text{posterior-PRP}}(c_0) &= \mathbb{P}(T' \geq T \mid \hat{\boldsymbol{\beta}}, R_0) && \text{(S11)} \\
&= \mathbb{E}_T \mathbb{P}(\chi^2_m - c_0 \geq T - c_0 \mid \hat{\boldsymbol{\beta}}, R_0) && \text{(S12)} \\
&= \int_{-\infty}^{\infty} [1 - F_{\chi^2_m}(s + c_0)] f_{\chi^2_1}(s) ds, && \text{(S13)}
\end{aligned}
$$

where $F_{\chi^2_m}(t)$ and $f_{\chi^2_1}(t)$ are the CDF and PDF of the chi-squared distribution with m and 1 degrees of freedom, respectively.

D.3 Special Case: Equal Effect Sizes

In the case where all observed effect sizes are equal

$$\hat{\beta}_1 = \ldots = \hat{\beta}_m,$$

then $c_0 = \sum_{j=1}^m w_j(\hat{\beta}_j - \mu_{\bar{\beta}})^2 = 0$. The test statistic is simplified to $T \sim \chi^2_1$, and the replicated statistic $T' \sim \chi^2_m$.

We first introduce some notation. Let $I_{d_1 t/(d_1 t + d_2)}\left(\frac{d_1}{2}, \frac{d_2}{2}\right) = \mathbb{P}(F_{d_1,d_2} \leq t)$, where F_{d_1,d_2} denote F-distribution with d_1 and d_2 degrees of freedom. $I_x(a,b)$ is also called regularized incomplete beta function

$$I_x(a,b) = \frac{\int_0^x t^{a-1}(1-t)^{b-1} dt}{\int_0^1 t^{a-1}(1-t)^{b-1} dt} = \frac{B_x(a,b)}{B(a,b)},$$

where $B(a,b)$ is the beta function. Now, we can compute $p_{\text{posterior-PRP}}(0)$ as

$$\begin{aligned}
p_{\text{posterior-PRP}}(0) &= \mathbb{P}\left(\chi^2_m \geq \chi^2_1\right) \\
&= \mathbb{P}\left(\frac{\chi^2_1/1}{\chi^2_m/m} \leq m\right) \\
&= \mathbb{P}\left(F_{1,m} \leq m\right) \\
&= I_{\frac{1}{2}}\left(\frac{1}{2}, \frac{m}{2}\right).
\end{aligned}$$

Now we will prove that $p_{\text{posterior-PRP}}$ is an increasing function with respect to m. By performing the substitution $t = \sin^2\theta$, $\theta \in [0, \pi/2]$, we can get another commonly used form of $I_{\frac{1}{2}}\left(\frac{1}{2}, \frac{m}{2}\right)$, that is,

$$\begin{aligned}
I_{\frac{1}{2}}\left(\frac{1}{2}, \frac{m}{2}\right) =& \frac{\int_0^{\frac{\pi}{4}} \cos^{m-1}\theta d\theta}{\int_0^{\frac{\pi}{2}} \cos^{m-1}\theta d\theta} \\
=& \frac{\int_0^{\frac{\pi}{4}} \cos^{m-1}\theta d\theta}{\int_0^{\frac{\pi}{4}} \cos^{m-1}\theta d\theta + \int_{\frac{\pi}{4}}^{\frac{\pi}{2}} \cos^{m-1}\theta d\theta} \\
=& \frac{1}{1 + \frac{\int_{\frac{\pi}{4}}^{\frac{\pi}{2}} \cos^{m-1}\theta d\theta}{\int_0^{\frac{\pi}{4}} \cos^{m-1}\theta d\theta}}.
\end{aligned}$$

In $[0, \pi/2]$, $\cos\theta$ is a continuous monotonic decreasing function taking values in $[0,1]$. As m increases, $\cos^{m-1}\theta$ becomes smaller, and the speed that $\cos^{m-1}\theta$ converges to 0 is faster in $[\pi/4, \pi/2]$ than that in $[0, \pi/4]$. Therefore, $I_{\frac{1}{2}}\left(\frac{1}{2}, \frac{m}{2}\right)$ is a continuous monotonic increasing function of m. And since $I_{\frac{1}{2}}\left(\frac{1}{2}, \frac{m}{2}\right)$ is a CDF, it naturally has an upper bound of 1. Therefore, we have

$$\lim_{m\to\infty} I_{\frac{1}{2}}\left(\frac{1}{2}, \frac{m}{2}\right) = 1.$$

D.3.1 Example

Some properties we need are as follows

$$
\begin{aligned}
I_x(a, 1) &= x^a, \\
I_x(a, b+1) &= I_x(a, b) + \frac{x^a(1-x)^b}{bB(a, b)}, \\
B(a, 1-a) &= \frac{\pi}{\sin(\pi a)}, \qquad a \notin \mathbb{Z}.
\end{aligned}
$$

- For $m = 2$:

$$
I_{\frac{1}{2}}\left(\frac{1}{2}, 1\right) = \left(\frac{1}{2}\right)^{\frac{1}{2}} \approx 0.7071.
$$

- For $m = 3$:

$$
\begin{aligned}
I_{\frac{1}{2}}\left(\frac{1}{2}, \frac{3}{2}\right) &= I_{\frac{1}{2}}\left(\frac{1}{2}, \frac{1}{2}\right) + \frac{\frac{1}{2}^{\frac{1}{2}}\frac{1}{2}^{\frac{1}{2}}}{\frac{1}{2}B(\frac{1}{2}, \frac{1}{2})} \\
&= \frac{B_{\frac{1}{2}}(\frac{1}{2}, \frac{1}{2})}{B(\frac{1}{2}, \frac{1}{2})} + \frac{1}{B(\frac{1}{2}, \frac{1}{2})} \\
&= \frac{\int_0^{\frac{1}{2}} t^{-\frac{1}{2}}(1-t)^{-\frac{1}{2}} dt}{\pi} + \frac{1}{\pi} \\
&= \left(2 \arcsin \sqrt{t}\ \Big|_0^{\frac{1}{2}}\right)/\pi + \frac{1}{\pi} \\
&= 2 \times \frac{\pi}{4}/\pi + \frac{1}{\pi} \approx 0.8183.
\end{aligned}
$$

- For $m = 4$:

$$
\begin{aligned}
I_{\frac{1}{2}}\left(\frac{1}{2}, 2\right) &= I_{\frac{1}{2}}\left(\frac{1}{2}, 1\right) + \frac{\frac{1}{2}^{\frac{1}{2}}\frac{1}{2}}{B(\frac{1}{2}, 1)} \\
&= \left(\frac{1}{2}\right)^{\frac{1}{2}} + \frac{\frac{1}{2}^{\frac{1}{2}}\frac{1}{2}}{\frac{\Gamma(1/2)\Gamma(1)}{\Gamma(1+1/2)}} \\
&= \left(\frac{1}{2}\right)^{\frac{1}{2}} + \frac{\frac{1}{2}^{\frac{1}{2}}\frac{1}{2}}{\frac{\Gamma(1/2)\Gamma(1)}{\frac{1}{2}\Gamma(1/2)}} \\
&= \left(\frac{1}{2}\right)^{\frac{1}{2}} \times \frac{5}{4} \approx 0.8804.
\end{aligned}
$$

- For $m = 5, 6, \ldots$, we can use recursive methods and property

$$I_x(a, b+1) = I_x(a, b) + \frac{x^a(1-x)^b}{bB(a, b)}.$$

D.4 Upper bound of $p_{\text{posterior-PRP}}$

$$\begin{aligned} p_{\text{posterior-PRP}}(c_0) &= \int_{c_0}^{\infty} [1 - F_{\chi_m^2}(t)] f_{\chi_1^2}(t - c_0) dt \\ &= \int_0^{\infty} [1 - F_{\chi_m^2}(s + c_0)] f_{\chi_1^2}(s) ds. \end{aligned}$$

Now, differentiate $p_{\text{posterior-PRP}}(c_0)$ with respect to c_0

$$\begin{aligned} \frac{dp_{\text{posterior-PRP}}(c_0)}{dc_0} &= \frac{d}{dc_0} \int_0^{\infty} \left[1 - F_{\chi_m^2}(s + c_0)\right] f_{\chi_1^2}(s)\ ds \\ &= \int_0^{\infty} \frac{d}{dc_0} \left[1 - F_{\chi_m^2}(s + c_0)\right] f_{\chi_1^2}(s)\ ds \\ &= -\int_0^{\infty} f_{\chi_m^2}(s + c_0) f_{\chi_1^2}(s)\ ds \leq 0. \end{aligned}$$

The last inequality comes from the fact PDFs $f_{\chi_m^2}(s + c_0), f_{\chi_1^2}(s)$ are always non-negative. This implies that $p_{\text{posterior-PRP}}(c_0)$ is a non-increasing function of c_0.

The maximum value of $p_{\text{posterior-PRP}}(c_0)$ occurs at $c_0 = 0$. Therefore,

$$p_{\text{posterior-PRP}}(c_0) \leq p_{\text{posterior-PRP}}(c_0)\Big|_{c_0=0} = I_{\frac{1}{2}}\left(\frac{1}{2}, \frac{m}{2}\right).$$

References

C. B. Begg and M. Mazumdar. Operating characteristics of a rank correlation test for publication bias. *Biometrics*, 50(4):1088–1101, 1994.

W. G. Cochran. The comparison of percentages in matched samples. *Biometrika*, 37(3): 256–266, 1950.

M. Egger, G. D. Smith, M. Schneider, and C. Minder. Bias in meta-analysis detected by a simple, graphical test. *British Medical Journal*, 315(7109):629–634, 1997.

W. K. Hastings. Monte carlo sampling methods using markov chains and their applications. *Biometrika*, 57(1):97–109, 1970.

A. Hróbjartsson and P. C. Gøtzsche. Placebo interventions for all clinical conditions. *Cochrane Database of Systematic Reviews*, 2010(2):CD003974, 2010.

C. J. Liu and N. K. Latham. Progressive resistance strength training for improving physical function in older adults. *Cochrane Database of Systematic Reviews*, 2009(3):CD002759, 2009.

N. Metropolis, A. W. Rosenbluth, M. N. Rosenbluth, A. H. Teller, and E. Teller. Equation of state calculations by fast computing machines. *Journal of Chemical Physics*, 21(6): 1087–1092, 1953.

K. D. Patil. Cochran's q test: Exact distribution. *Journal of the American Statistical Association*, 70(349):186–189, 1975.

L. F. Stead, R. Perera, C. Bullen, D. Mant, J. Hartmann-Boyce, K. Cahill, and T. Lancaster. Nicotine replacement therapy for smoking cessation. *Cochrane Database of Systematic Reviews*, 2012(11):CD000146, 2012.
